# TNFR1 signaling promotes pancreatic tumor growth by limiting dendritic cell number and function

**DOI:** 10.1016/j.xcrm.2024.101696

**Published:** 2024-08-22

**Authors:** Muhammad S. Alam, Matthias M. Gaida, Hagen R. Witzel, Shizuka Otsuka, Aamna Abbasi, Theresa Guerin, Abdalla Abdelmaksoud, Nathan Wong, Margaret C. Cam, Serguei Kozlov, Jonathan D. Ashwell

**Affiliations:** 1 Laboratory of Immune Cell Biology, Center for Cancer Research, National Cancer Institute, National Institutes of Health, Bethesda, MD 20892, USA; 2 Department of Integrative Immunobiology, Duke University, Durham, NC 27708, USA; 3 Institute of Pathology, University Medical Center Mainz, JGU-Mainz, 55131 Mainz, Germany; 4 TRON, Translational Oncology at the University Medical Center, JGU-Mainz, 55131 Mainz, Germany; 5 Research Center for Immunotherapy, University Medical Center Mainz, JGU-Mainz, 55131 Mainz, Germany; 6 Collaborative Bioinformatics Resource (CCBR), Center for Cancer Research, National Cancer Institute, National Institutes of Health, Bethesda, MD 20892, USA; 7 Advanced Biomedical Computational Science, Frederick National Laboratory for Cancer Research, Frederick, MD 21702, USA; 8 Center for Advanced Preclinical Research, Frederick National Laboratory for Cancer Research, Frederick, MD 21707, USA

**Keywords:** inflammation, TNFR1, TNF-α, pancreatic adenocarcinoma, PDAC, KPC mice, dendritic cells, apoptosis

## Abstract

Pancreatic adenocarcinoma (PDAC) is one the most intractable cancers, in part due to its highly inflammatory microenvironment and paucity of infiltrating dendritic cells (DCs). Here, we find that genetic ablation or antibody blockade of tumor necrosis factor receptor 1 (TNFR1) enhanced intratumor T cell activation and slowed PDAC growth. While anti-PD-1 checkpoint inhibition alone had little effect, it further enhanced intratumor T cell activation in combination with anti-TNFR1. The major cellular alteration in the tumor microenvironment in the absence of TNFR1 signaling was a large increase in DC number and immunostimulatory phenotype. This may reflect a direct effect on DCs, because TNF induced TNFR1-dependent apoptosis of bone-marrow-derived DCs. The therapeutic response to anti-TNFR1 alone was superior to the combination of DC-activating agonistic anti-CD40 and Flt3 ligand (Flt3L). These observations suggest that targeting TNFR1, perhaps in concert with other strategies that promote DC generation and mobilization, may have therapeutic benefits.

## Highlights

• TNF has a pro-tumorigenic role in PDAC by limiting intratumor DC number and function
• TNFR1 blockade increased DC-dependent anti-tumor immunity and inhibited tumor growth
• Signaling via TNFR1 induced DC apoptosis *in vitro*

## Graphical abstract

**Figure:**
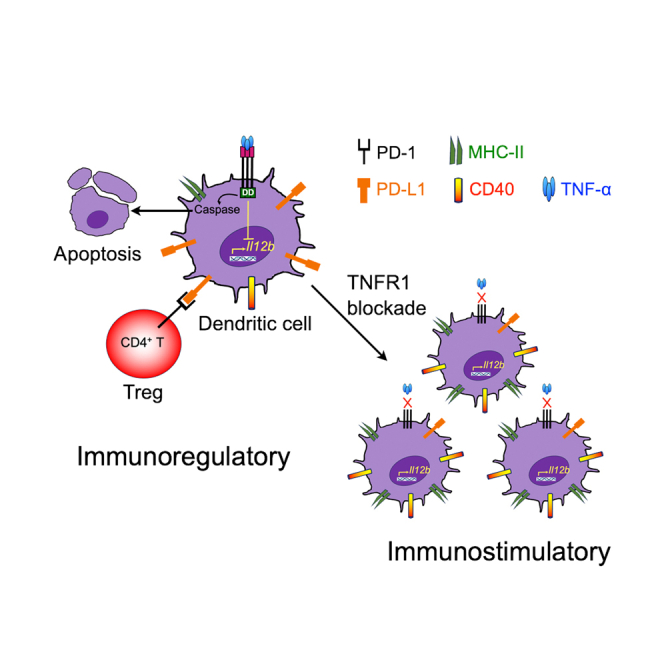

## Teaser

Alam et al. found that in pancreatic ductal adenocarcinomas, the pro-inflammatory cytokine TNF-α signals via TNFR1, its major receptor, to prevent the infiltration and activation of antigen-presenting dendritic cells. These effects were prevented by TNFR1 blockade, resulting in increased T cell-mediated anti-tumor immunity and inhibition of tumor progression.

## Introduction

Pancreatic ductal adenocarcinoma (PDAC) is the fourth leading cause of cancer-related deaths in the Western world and is predicted to be the second leading cause of cancer deaths by 2030.^1^ PDAC is highly resistant to conventional therapeutic approaches and relatively refractory to immune checkpoint inhibition.^2^ The nature of PDAC is complex, and its inflammatory tumor microenvironment is a major source of pro-tumorigenic cytokines and chemokines that lead to tumor initiation, progression, and metastasis.^1^^,^^3^ Features of the inflammatory microenvironment that have been reported to contribute to tumor progression and therapeutic resistance are hypovascularity, a dense fibrotic stroma with cancer-associated fibroblasts (CAFs),^3^^,^^4^ infiltration with myeloid-derived suppressor cells (MDSCs)^5^ and tumor-associated macrophages,^6^^,^^7^ ineffective cytolytic CD8^+^ T cells due to exhaustion,^8^^,^^9^ and increased numbers of CD4^+^ regulatory T (Treg) cells.^10^^,^^11^ Another mechanism of immune escape is the paucity of tumor-infiltrating dendritic cells (DCs), which are essential for effective anti-tumor T cell responses, in mice^12^ and humans.^13^^,^^14^ Tumor DCs are heterogeneous populations involved in different aspects of T cell responses. *Batf3/Irf8*-dependent type 1 DCs (DC1s) are important in the cross-presentation of tumor antigens to CD8^+^ T cells, *Irf4*-dependent type 2 DCs (DC2s) prime and polarize antigen-specific CD4^+^ T helper (Th) cells,^15^ *Irf4*-dependent monocytic DCs (moDCs) are capable of CD8^+^ T cell cross-priming^16^ and prime CD8^+^ T cells, regulatory DCs (mregDCs) produce large amounts of interleukin-12 (IL-12) ubiquitously,^17^ and plasmacytoid DCs (pDCs) can produce large quantities of type I interferons.^18^ When activated, DCs increase expression of a number of functionally important molecules. Upregulation of major histocompatibility complex-II (MHC-II), CD80, and CD86 results in better antigen presentation and T cell costimulation.^19^ Upregulated CD40 binds to CD40L on CD4^+^ T cells and enhances DC production of critical cytokines such as IL-12.^20^ In human PDAC, the levels of circulating DCs positively correlate with survival.^13^^,^^14^ Furthermore, higher numbers of infiltrating DCs,^13^^,^^14^ in particular DC1s, DC2s, and pDCs, have been correlated with better survival.^21^ Therefore, strategies to increase DC number in PDAC would be expected to have therapeutic benefits.

Tumor necrosis factor (TNF), an inflammatory cytokine involved in various autoimmune and inflammatory diseases and cancer,^22^ has been shown to enhance PDAC growth.^4^^,^^22^^,^^23^^,^^24^ Although TNF is secreted by many cells, including in some cases tumor cells themselves, TNF produced by CD4^+^ T cells was shown to accelerate the growth of a PDAC cell line,^24^ and TNF produced by macrophages^25^ and neutrophils^26^ was shown to play a pro-tumorigenic role in an oncogene-driven PDAC mouse model.

TNF binds to and signals via two different receptors, the ubiquitously expressed death-domain-containing TNF receptor 1 (TNFR1) and the tissue-restricted TNF receptor 2 (TNFR2).^27^ Signaling via TNFR1 results in a variety of distinct biological responses, including the induction of pro-inflammatory cytokine secretion via activation of mitogen-activated protein kinases (MAPKs) and canonical nuclear factor κB (NF-κB), caspase-mediated apoptosis, and RIP1/RIP3/MLKL-dependent necroptosis.^27^ Unlike TNFR1, signaling via TNFR2 does not induce cell death and upregulates NF-κB via the noncanonical pathway.^28^^,^^29^ Relatively little is known about the effect of selective TNFR signaling on PDAC-infiltrating hematopoietic cell subsets.

In this study, we explored the molecular and cellular mechanisms by which TNF promotes PDAC progression. Using an oncogenic K-ras-driven PDAC mouse model (KPC mice), we found that signaling via TNFR1, but not TNFR2, in cells of the immune system profoundly limits tumor-infiltrating DC number and function, and its abrogation by genetic or therapeutic means led to a marked increase in DC infiltration and activation, and a more effective anti-tumor response.

## Results

### Reduced KPC tumor growth in TNFR1-deficient mice

**Figure 1:**
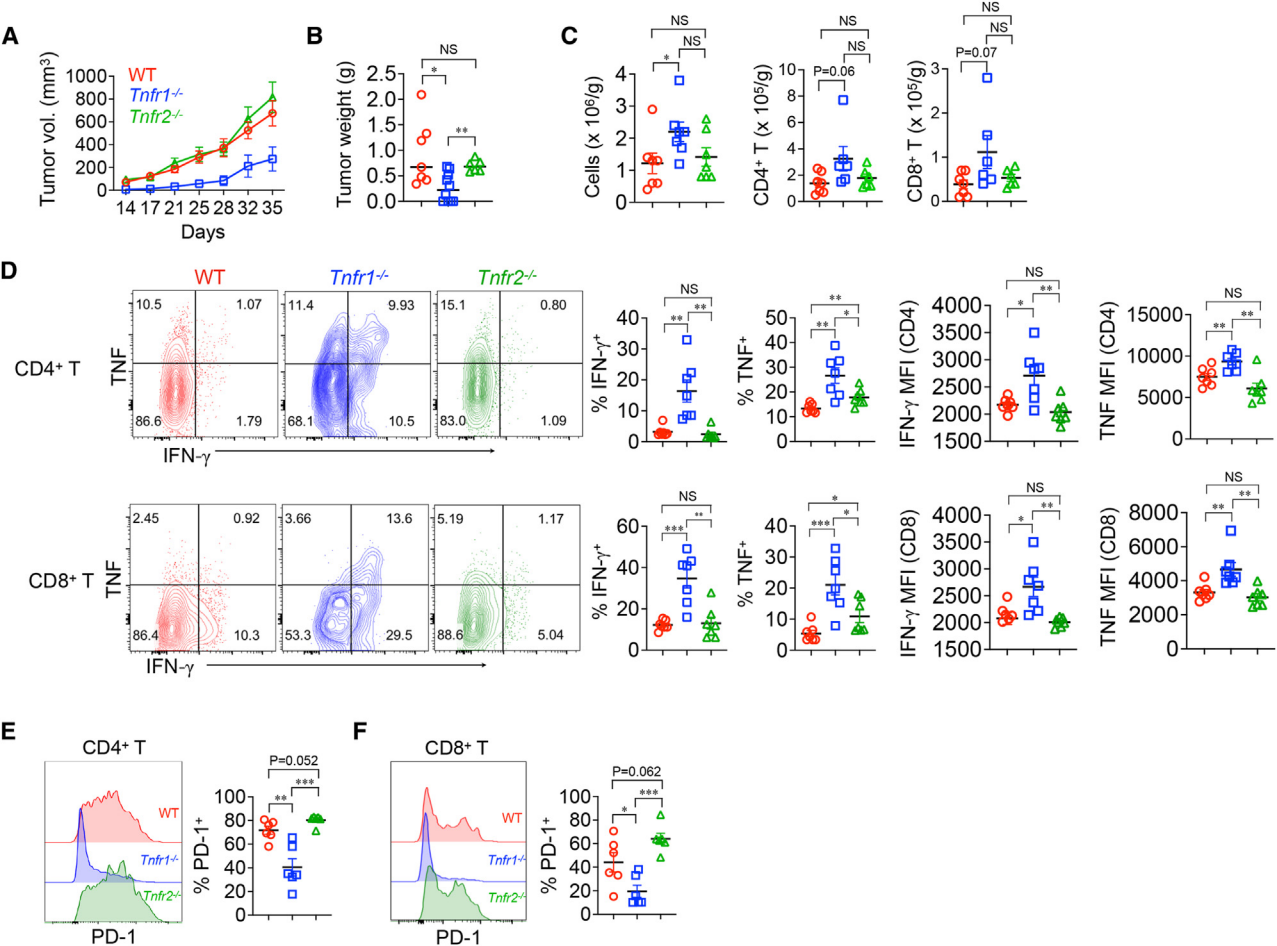
Reduced PDAC growth and increased intratumor T cell activation in TNFR1-deficient mice (A–F) KPC cells were subcutaneously implanted in the flank of WT (*n* = 7), *Tnfr1*^*−/−*^ (*n* = 9), and *Tnfr2*^*−/−*^ (*n* = 6) mice. Tumor volumes were measured over time (A) and were harvested for weighing (B) and isolation of infiltrating cells (C). Infiltrating cells were stimulated with ionomycin in the presence of monensin, and IFN-γ and TNF production in CD4^+^ (Thy1.2^+^CD4^+^) and CD8^+^ (Thy1.2^+^CD8β^+^) T cells were determined by intracellular staining and flow cytometry (D). PD-1 expression on CD4^+^ (TCRβ^+^CD4^+^) (E) and CD8^+^ (TCRβ^+^CD8^+^) (F) was measured by flow cytometry. The gating strategy for flow cytometry analyses is shown in Figure S1. ∗*p* < 0.05, ∗∗*p* < 0.01, ∗∗∗*p* < 0.001. NS, not significant.

Although TNF has been shown to enhance PDAC growth,^4^^,^^22^^,^^24^ the underlying mechanism is unclear. To investigate this question, we used a K-ras-driven genetically modified model of PDAC that mimics the clinical disease. Initial studies were performed with a cell line generated from primary KPC mouse tumors. Tumor cells were injected subcutaneously into wild-type (WT), TNFR1-deficient (*Tnfr1*^*−/−*^), and TNFR2-deficient (*Tnfr2*^*−/−*^) mice (Figures 1A and 1B). Cancer progression, as measured by tumor volume over time and tumor weight at the 35-day experimental endpoint, was similar between WT and *Tnfr2*^*−/−*^ mice, as were the numbers of tumor-infiltrating immune cells (Figure 1C). The rate of tumor progression in TNFR1-deficient mice, however, was clearly diminished and was accompanied by an increase in the number of tumor-infiltrating cells, including T cells. Both the percentage of infiltrating CD4^+^ and CD8^+^ T cells that produced IFN-γ- and/or TNF and the amount of each produced per cell (measured by mean fluorescence intensity [MFI]) were higher in *Tnfr1*^*−/−*^ animals compared to the other genotypes (Figures 1B–1D). In addition, expression of the T cell programmed death 1 (PD-1) exhaustion marker was reduced on both CD4^+^ and CD8^+^ T cells from *Tnfr1*^*−/−*^ mice compared to WT or *Tnfr2*^*−/−*^ T cells (Figures 1E and 1F). The slower tumor growth in *Tnfr1*^*−/−*^ mice was not cell line specific, as the growth of the Panc02 murine pancreatic cell line was also reduced in *Tnfr1*^*−/−*^ mice (Figure S1B), which was accompanied by increased numbers of tumor-infiltrating immune cells and higher percentages of IFN-γ-producing CD4^+^ and CD8^+^ T cells (Figures S1C and S1D). Therefore, the reduced tumor growth in *Tnfr1*^*−/−*^ mice correlated with more infiltrating and activated effector T cells.

### Antibody blockade of TNFR1 with or without PD-1 checkpoint blockade

**Figure 2:**
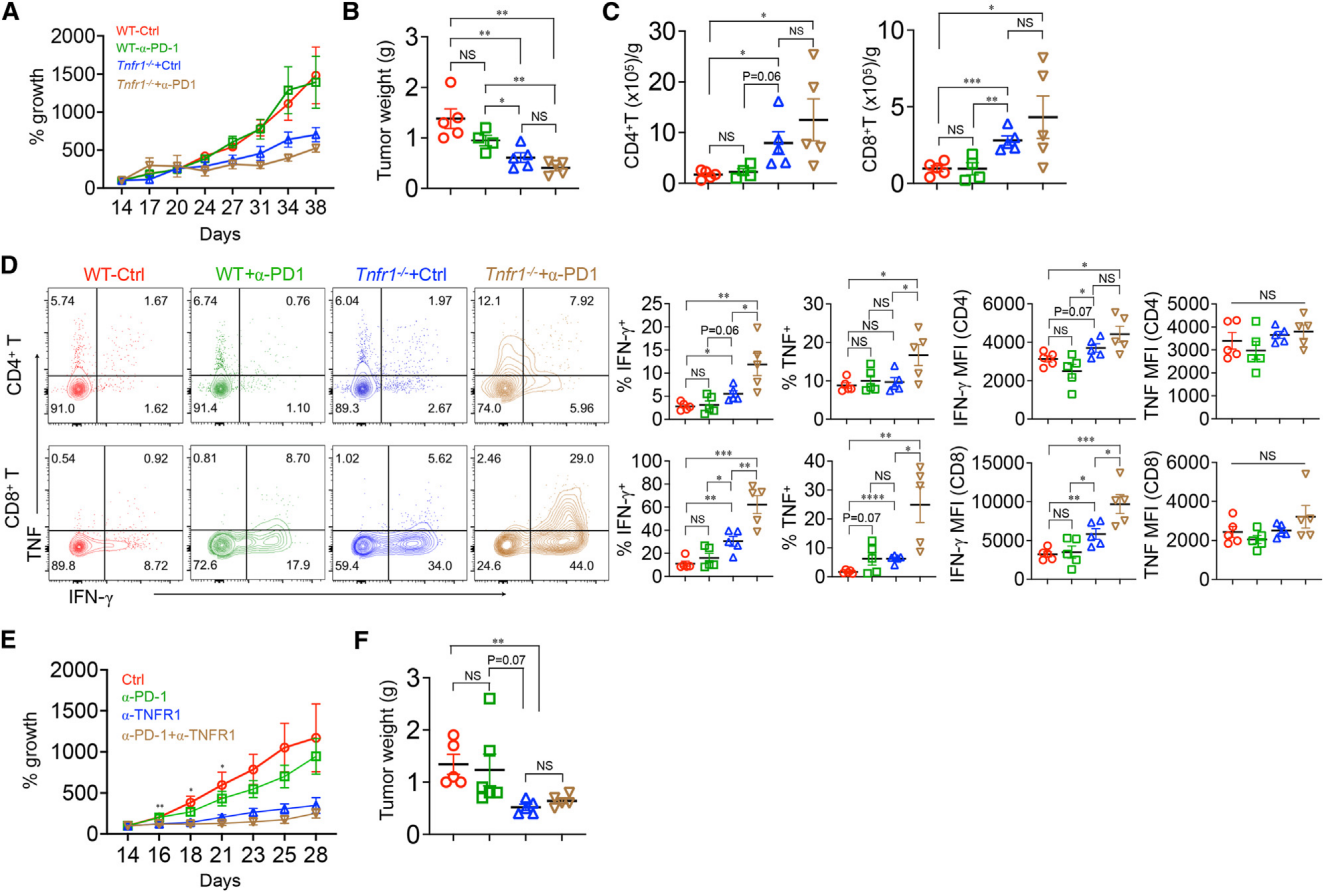
Treatment of subcutaneously injected KPC cells (A–D) KPC cells were subcutaneously implanted in WT or TNFR1-deficient mice, and 2 weeks later, the tumors were injected with control (WT-ctrl, *n* = 5; *Tnfr1*^*−/−*^-ctrl, *n* = 5) or anti-PD-1 antibody (α-PD-1) (WT-α-PD-1, *n* = 4; *Tnfr1*^*−/−*^-α-PD-1, *n* = 5), and tumor progression was monitored. Growth is shown as the percent increase from day 14. On day 38, the tumors were weighed (B) and tumor-infiltrating T cells enumerated (C) and stimulated with PMA/ionomycin to induce cytokine production (D). (E and F) WT mice were injected with KPC cells as in A. After 14 days, the tumors were injected with either control (*n* = 6), anti-PD-1 (α-PD-1) (*n* = 6), anti-TNFR1 (α-TNFR1) (*n* = 6), or both α-PD-1 and α-TNFR1 antibodies (α-PD-1 + α-TNFR1) (*n* = 5) (E). Growth is shown as the percent increase from day 14. Tumor weight was measured on day 28 (F). ∗*p* < 0.05, ∗*p* < 0.01, ∗∗∗*p* < 0.001, ∗∗∗∗*p* < 0.0001. NS, not significant.

Although PDAC is relatively resistant to immune checkpoint blockade,^2^ we asked if anti-PD-1 could further increase the intratumor T cell activity resulting from TNFR1 deficiency. Treatment of KPC cell-inoculated WT mice with anti-PD-1 alone had only a small effect on growth (Figures 2A and 2B). Tumor growth was substantially slower in *Tnfr1*^*−/−*^ mice, and tumor weight was modestly but not statistically significantly decreased with the addition of anti-PD-1. The accumulation of tumor-infiltrating CD4^+^ and CD8^+^ T cells reflected the differences in growth (Figure 2C). Treatment of WT mice with anti-PD-1 had no effect on infiltrating T cell numbers compared to control. There was a 3- to 4-fold increase in the number of tumor-infiltrating CD4^+^ and CD8^+^ T cells in *Tnfr1*^*−/−*^ mice, which was not demonstrably increased by the addition of anti-PD-1. The fraction of T cells producing IFN-γ and TNF after stimulation with phorbol 12-myristate 13-acetate (PMA) and ionomycin was similar between control and anti-PD-1-treated WT animals but was increased by anti-PD-1 treatment in *Tnfr1*^*−/−*^ mice (Figure 2D). In contrast to the relatively little change in T cell numbers, the addition of anti-PD-1 to TNFR1 knockout (KO) mice resulted in large increases in the percentage of CD4^+^ and, in particular, CD8^+^ T cells that produced IFN-γ and TNF and the amounts of IFN-γ produced per cell.

The effect of antibody-mediated blockade of TNFR1 in WT mice was similar to that observed in *Tnfr1*^*−/−*^ mice. Treatment with anti-PD-1 alone had a small effect on tumor progression, whereas treatment with anti-TNFR1 caused a substantial inhibition of tumor growth (Figure 2E). The effect of adding anti-PD-1 was difficult to assess because anti-TNFR1 alone was so effective. The accumulation of tumor-infiltrating T cells varied accordingly, being much higher in mice treated with anti-TNFR1 with or without anti-PD-1 (Figure S2A). As seen with tumors in *Tnfr1*^*−/−*^ mice, blockade of both TNFR1 and PD-1 signaling resulted in large increases in the number of T cells producing IFN-γ and TNF, as well as the amount produced per cell (Figure S2B). Taken together, although the addition of PD-1 blockade to anti-TNFR1 had little enhancing effect on the number of tumor-infiltrating T cells, the combination resulted in a robust increase in their production of effector cytokines.

### Lack of T cell TNFR1 signaling is insufficient to enhance anti-tumor immunity

The increase in tumor-infiltrating T cell activation and function caused by loss of TNFR1 signaling led us to speculate that the T cells were a direct target of TNF. Tο determine if the effect of TNF was T cell intrinsic, lymphocyte-deficient *RAG2*^*−/−*^ mice were reconstituted with WT, *Tnfr1*^*−/−*^, or *Tnfr2*^*−/−*^ T cells and inoculated with KPC cells 2 weeks later. Compared to mice that did not receive any T cells, tumor growth was not inhibited but actually enhanced, especially late in the response of mice that received *Tnfr2*^*−/−*^ T cells (Figure S2C). Enhanced growth may reflect the effect of T cell-derived TNF, which has been shown to enhance Panc02 PDAC tumor growth.^24^ Similar results were obtained with transferred purified CD8^+^ T cells (Figure S2D). These results suggest that signaling via T cell TNFR1 alone was not sufficient to enhance an anti-tumor immune response.

### TNFR1-derived signals impair DC-dependent anti-tumor immune responses

**Figure 3:**
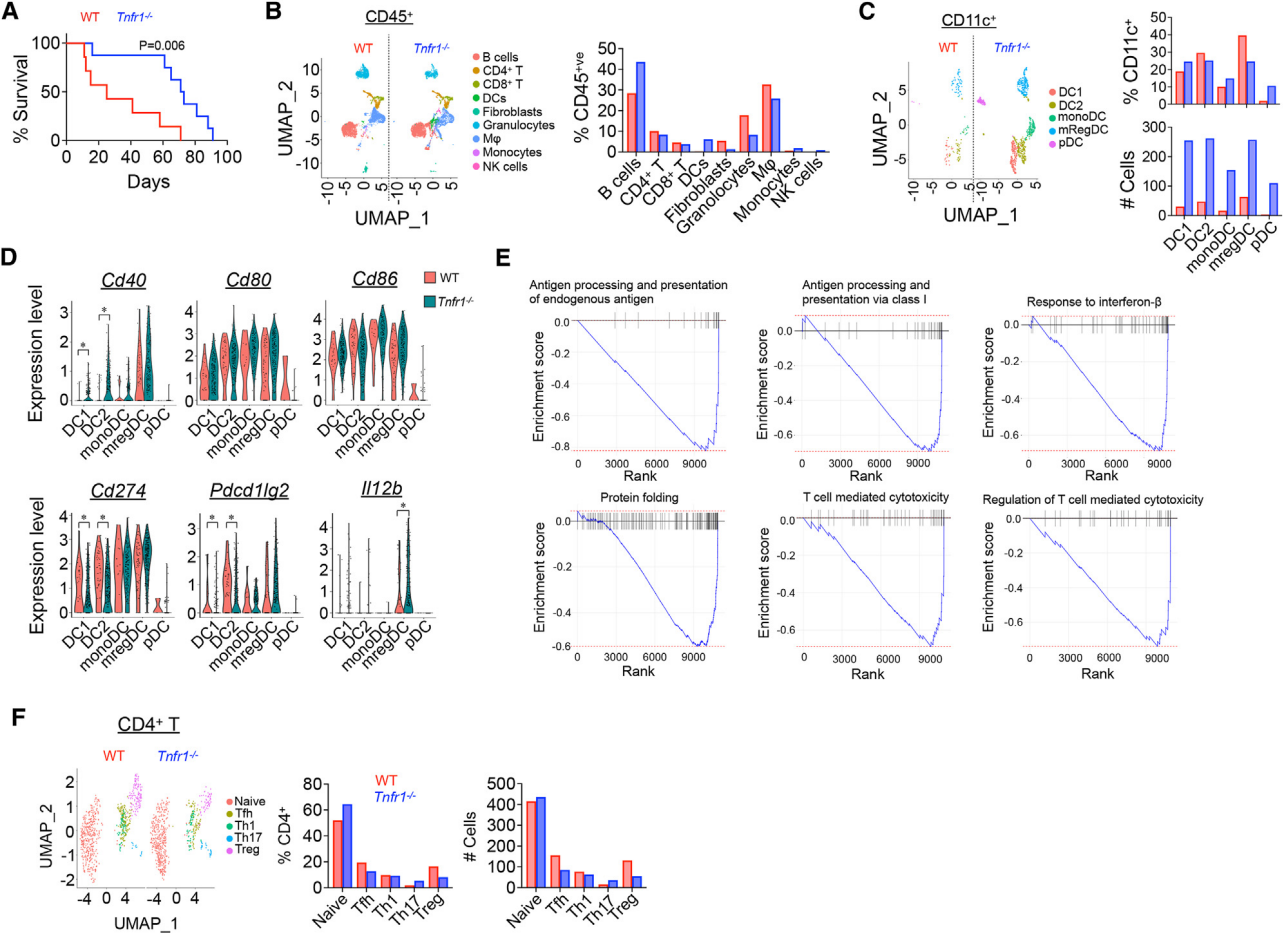
Single-cell sequencing of tumor-infiltrating hematopoietic cells in radiation bone marrow chimeras (A–F) KPC mice were lethally irradiated and reconstituted with either WT (*n* = 7) or TNFR1-deficient (*n* = 8) bone marrow. Survival of mice after transfer (day 0) is shown in (A). Single-cell RNA-seq was performed with infiltrating CD45^+^ cells from ∼300 mm^3^ tumors (B) and DCs (C). Violin plots of the indicated gene expression in DCs (D). GSEA of WT DC2s compared to TNFR1-deficient DC2s. Downward curves represent genes enriched in TNFR1-deficient DCs (E). Uniform manifold approximation and projection (UMAP) plot of CD4^+^ tumor-infiltrating T cells (F). ∗*p* < 0.05.

To determine whether TNFR1 signaling in hematopoietic cells was responsible for suppressing anti-tumor immunity, lethally irradiated 8-week-old KPC mice, which spontaneously develop multifocal K-ras-driven PDAC at approximately 10–14 weeks of age,^30^ were reconstituted with WT or *Tnfr1*^*−/−*^ bone marrow (BM). Notably, mice receiving TNFR1-deficient BM survived approximately 3 times longer than mice receiving WT BM (50% survival: WT, 22 days; *Tnfr1*^−/−^, 72 days) (Figure 3A). Tumor-infiltrating cells were isolated from another group of irradiated BM chimeras 24 days after reconstitution and sort-purified into two groups, CD45^+^ (all hematopoietic cells) and CD45^+^CD11c^+^ (DCs). Single-cell RNA sequencing (scRNA-seq) analysis of CD45^+^ cells found that the immune cell composition was broadly similar between the two groups, with comparable percentages of CD4^+^ and CD8^+^ T cells (Figure 3B). There was a small increase in the fraction of B cells with offsetting decreases on the percentages of granulocytes and macrophages in the tumors of mice receiving *Tnfr1*^*−/−*^ BM. A notable exception was DCs, which were almost undetectable in WT but constituted approximately 5% of the total in mice receiving TNFR1-deficient BM. Further analysis of sorted CD45^+^CD11c^+^ DCs found that all subsets were increased in the absence of TNFR1 (Figure 3C). There was also evidence of an enhancement in their ability to stimulate T cells (gene signatures of each DC subset, DC1 [*Xcr1*, *Wdfy4*, *Cadm1*, etc.], DC2 [*Cd14*, *Sirpa*], moDC [*Adgre1*, *Ly6c6g*, etc.], mregDC [*Ccr7*, *Birc3*, *Tmem176a*, etc.], and pDC [*Siglech*, *Sell*, *Mpeg1*, *Bcl11a*, etc.] are shown in Figure S3A). TNFR1-deficient DC1s and DC2s had elevated expression of *Cd40*, a cell surface receptor whose engagement leads to IL-12 production^31^ and correspondingly higher expression of *Il12* from mregDCs (Figure 3D). In addition, TNFR1-deficient DC1s and DC2s had much lower expression of *Cd274* and *Pdcd1Ig2* (encoding PD-L1 and PD-L2, respectively, ligands for T cell PD-1) (Figure 3D). Gene set enrichment analysis (GSEA) identified elevated expression of genes in many pathways related to antigen presentation in *Tnfr1*^*−/−*^ DCs (Figures 3E, S3B, and S3C). The numbers of immune-suppressing Tregs were approximately 2-fold higher and effector Th17 cells were 3-fold lower in tumors reconstituted with WT compared to TNFR1-deficient BM (Figures 3F and S3D). These data indicate that a major contributor to the aggressive nature of oncogene-driven KPC PDAC is local immune dysfunction caused by TNFR1-dependent suppression of DC number and function.

### TNFR1 blockade increases intratumor DC number but not activation in the absence of T cells

**Figure 4:**
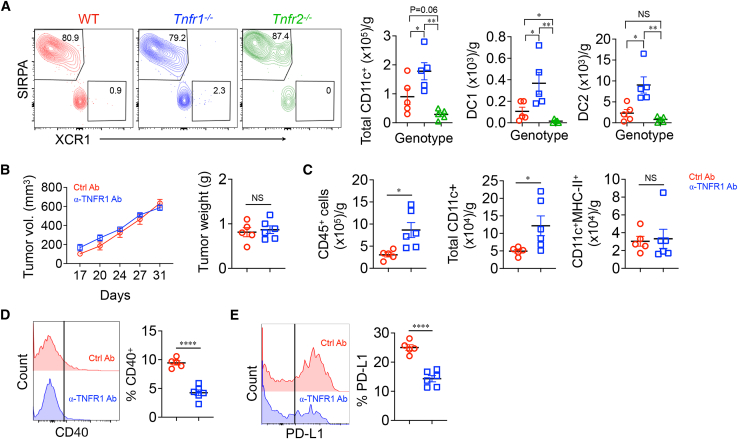
Effect of TNFR1 blockade on KPC tumors in RAG2-deficient mice (A) KPC cells were subcutaneously implanted in the flank of WT (*n* = 5), *Tnfr1*^−/−^ (*n* = 5), and *Tnfr2*^−/−^ (*n* = 5) mice. Tumor-infiltrating DCs were analyzed. (B–E) KPC cells were subcutaneously implanted in *RAG*2^−/−^ mice, allowed to grow for 17 days, and then treated with control (*n* = 5) or anti-TNFR1 antibody (*n* = 6) every 3 days. Tumor volumes were measured over time (B, left) and tumors removed on day 31 for weighing (B, right) and further analysis (C–E). ∗*p* < 0.05, ∗∗*p* < 0.01, ∗∗∗*p* < 0.001, ∗∗∗∗*p* < 0.0001. NS, not significant.

Because of the similar nature of the autochthonous and subcutaneous KPC mouse model of PDAC, we investigated whether single-cell sequence data of higher DC infiltration were true for the subcutaneous model. Total CD11c^+^ cells including DC1s (CD11c^+^MHC-II^hi^XCR1^+^) and DC2s (CD11c^+^MHC-II^hi^SIRPA^+^) were higher in TNFR1 KO mice compared to WT and TNFR2 KO mice (Figure 4A, gating strategy; Figure S4A). TNFR2 KO mice had the lowest number of total CD11C^+^ cells with a few or no DC1s and substantially reduced number of DC2s. Similar results were obtained when different markers were used to identify classical DCs (cDCs, CD11b^+^Ly6c^−^CD11c^+^MHC-II^hi^CD24^+^), DC1s (CD103^+^), and DC2s (CD11b^+^)^32^ (gating strategy: Figures S7B and S7C). Similar results were obtained when different markers were used to identify cDCs ( CD11b^+^Ly6c^−^CD11c^+^MHC-II^hi^CD24^+^), DC1s (CD103^+^), and DC2s (CD11b^+^)^32^ (gating strategy: Figures S4B and S4C). As similar data were obtained with either gating strategy, we used XCR1 expression as a DC1 and SIRPA as a DC2 marker in subsequent analyses.

Bilateral communication between DCs and T cells is required for effective anti-tumor responses.^20^^,^^31^ To determine how TNFR1 signaling affects intratumor DC number and activity in the absence of TNFR1 signaling, established subcutaneous KPC tumors in *RAG2*^−/−^ mice were treated with anti-TNFR1 or control antibodies. Unlike its effect in WT mice, anti-TNFR1 had no effect on tumor progression in the absence of T cells (Figures 4B and S4D). It did, however, increase the infiltration of CD45^+^ hematopoietic cells and DCs (Figure 4C). Notably, these CD11c^+^ DCs had an inactivated phenotype, being MHC-II^lo^, and fewer expressed CD40 or PD-L1 compared to sham-treated controls (Figures 4C–4E). Therefore, T cells are not required for the increased number of tumor-infiltrating DCs found in the absence of TNFR1 signaling but are required for intratumor DC activation and inhibition of tumor growth.

### Blockade of TNFR1 and PD-1 inhibits spontaneous PDAC growth

**Figure 5:**
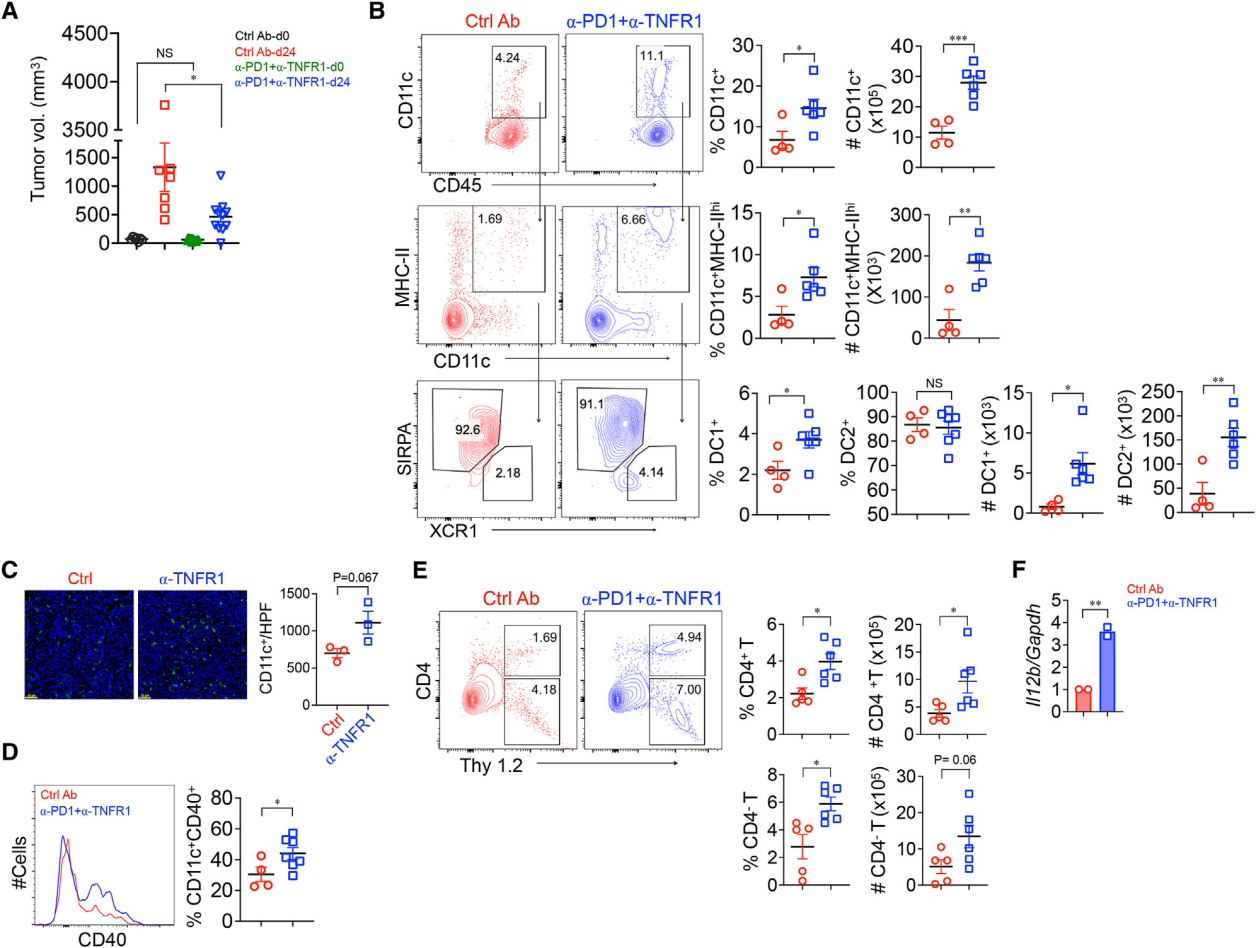
Simultaneous blockade of TNFR1 and PD-1 reduced KPC tumor growth KPC mice in which established pancreatic tumors were detected by ultrasound were treated with either control (*n* = 7) antibody or anti-TNFR1 plus anti-PD-1 antibody (*n* = 11). (A–D) Tumors were harvested (ctrl, *n* = 4; anti-TNFR1, *n* = 3; and anti-TNFR1 plus anti-PD-1, *n* = 6) at day 24, and measured tumor volume (A), infiltrating total DCs (B, C), activated DCs (CD11c^+^CD40^+^) (D), and T cells (E) were analyzed. In the figure shown in C, pancreatic tumor tissues were stained for CD11c (green color) and DAPI (blue). Histogram showed the number of CD11c-positive cells per high power field (HPF). (F) *Il12b* expression of purified DCs measured by quantitative real-time PCR. Each data point represents pooled DCs from 3 mice. ∗*p* < 0.05, ∗∗*p* < 0.01, ∗∗∗*p* < 0.001. NS, not significant.

To determine if the effects of TNFR1 blockade on subcutaneously implanted KPC cells could be extended to the naturally occurring disease, 7- to 8-week-old KPC mice were screened by ultrasound for the appearance of pancreatic tumors. Animals bearing tumors with volumes of 50–100 mm^3^ were randomized into two groups, one receiving control antibody and the other receiving anti-TNFR1 plus anti-PD-1. After 19–24 days, tumor volumes were re-evaluated by ultrasound. Tumors were subsequently removed and dissected, and infiltrating cells were isolated. Tumors were much smaller in mice receiving anti-TNFR1 plus anti-PD-1 compared to control (Figure 5A). This was associated with higher percentages and absolute numbers of infiltrating activated (B220^−^CD45^+^CD11c^+^ MHC-II^hi^) DCs, DC1s (CD11c^+^MHC-II^hi^XCR^+^), and CD11c^+^CCR7^+^ mregDCs (Figures 5B and S5A, gating strategy; Figures S5B and S5C). There was also an approximately 2-fold increase in the small number of tumor-infiltrating DC1s (CD11c^+^MHC-II^hi^XCR^+^). DC2s (CD11c^+^MHC-II^hi^SIRPA^+^) were the predominant subset in PDAC, and their number increased by approximately 3-fold in mice receiving anti-TNFR1 together with anti-PD-1. This increase in DC number was also observed by immunohistochemistry in tumor sections of KPC mice treated with anti-TNFR1 alone (Figure 5C). Notably, anti-TNFR1/PD-1 caused almost a doubling of the percentage of DCs expressing CD40, meaning that there was approximately a 6-fold expansion of CD40^+^ DCs (Figure 5D). Both the fraction and the actual number of infiltrating CD4^+^ T and CD8^+^ T cells were increased by anti-TNFR1/PD-1 (Figure 5E). Quantitative real-time PCR of DCs purified from KPC tumors found increased *Il12* in the treated group (Figure 5F), as had been observed in irradiated KPC mice that received *Tnfr1*^*−/−*^ BM (see Figure 3D). Taken together, the data demonstrate that anti-TNFR1/PD-1 increases the number and activation state of infiltrating DCs in K-ras-driven spontaneous tumors.

### Comparison of TNFR1 blockade versus Flt3L plus agonistic anti-CD40

**Figure 6:**
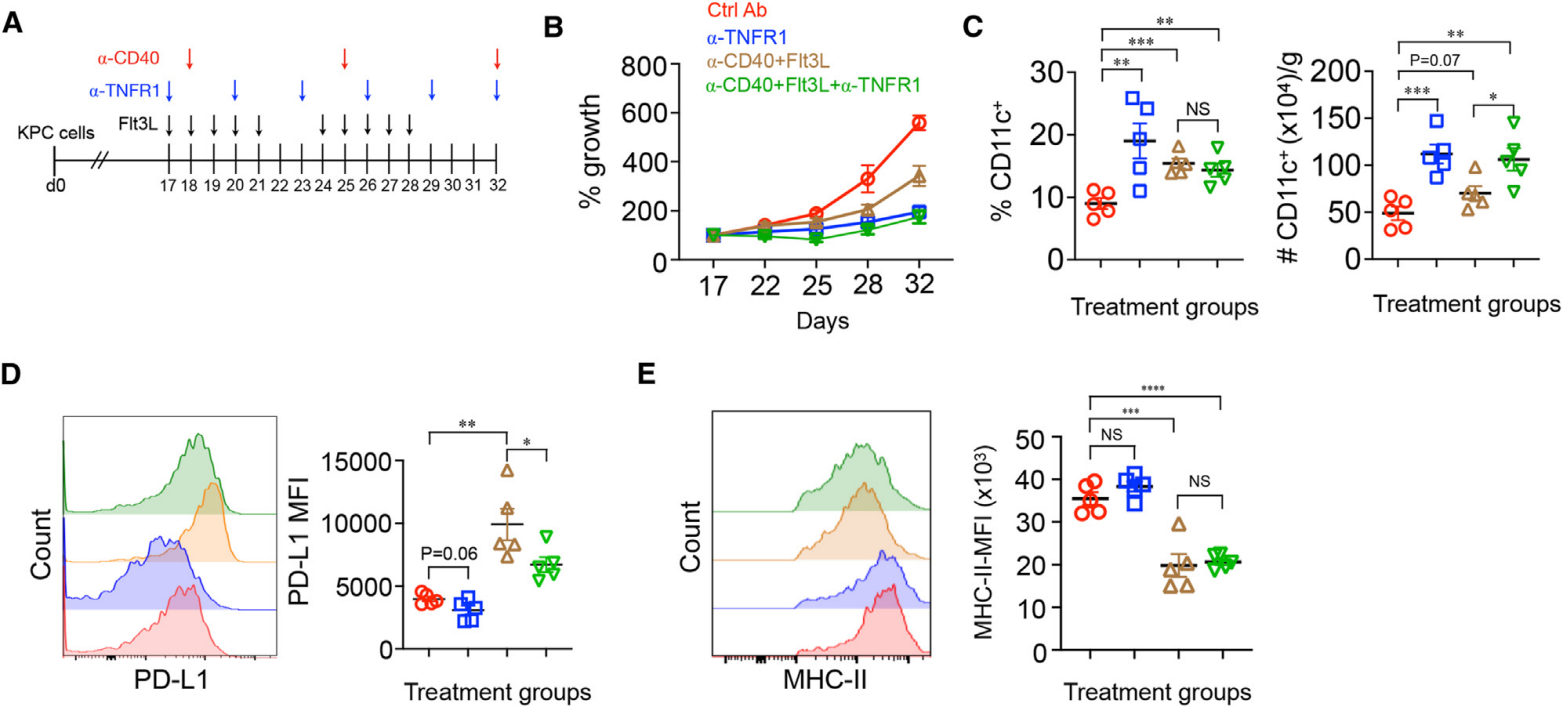
Comparative efficacy of TNFR1 blockade vs. Flt3L and agonistic anti-CD40 in treating subcutaneous KPC cell tumors (A–D) KPC cells were subcutaneously injected into WT or TNFR1-deficient mice and allowed to grow for 17 days. Schematic representation of therapeutic regimen is shown in (A), and the percent tumor growth since day 17 is shown in (B). On day 32, the percentage and number of tumor-infiltrating DCs (C) and their PD-L1 (D) and MHC-II expression (E) were measured. ∗*p* < 0.05, ∗∗*p* < 0.01, ∗∗∗*p* < 0.001, ∗∗∗∗*p <* 0.0001. NS, not significant.

A previous study found that the combination of Flt3 ligand (Flt3L) and agonistic anti-CD40 antibodies increased the percentage of intratumor DCs and prolonged survival in KPC mice.^12^ Because the effect of anti-TNFR1 is similar, we asked how they compared, alone or in combination. Established subcutaneous KPC cell tumors were treated with these regimens individually or in combination (Figure 6A). Anti-TNFR1 alone substantially reduced tumor growth compared to control (Figure 6B), which correlated positively with an increased percentage of intratumor DCs (Figures S6A and S6C, left panel) and number (Figure 6C, right panel). Flt3L plus agonistic anti-CD40 also increased DC numbers and slowed tumor growth, but to a lesser extent (Figures 6B and 6C). There was little difference between the response to all three reagents versus anti-TNFR1 alone. The predominant DC subset remained DC2s in all therapeutic arms (Figure S6B). Of note, whereas anti-TNFR1 inhibited DC PD-L1 expression, Flt3L and agonistic anti-CD40 resulted in an approximately 2.5-fold increase in cell surface levels (Figure 6D). Furthermore, the levels of MHC-II expressed on tumor-infiltrating DCs, a measure of their maturational and functional state, were much lower in anti-CD40+Flt3L-treated mice compared to control, whereas MHC-II levels on DCs from anti-TNFR1-treated mice were similar to if not greater than control mice (Figure 6E). Therefore, compared to anti-CD40+Flt3L, TNFR1 blockade was more effective in increasing the number of intratumor immunostimulatory DCs, which was reflected in better control of tumor growth.

### TNFR1 signaling results in apoptotic death of BMDCs

**Figure 7:**
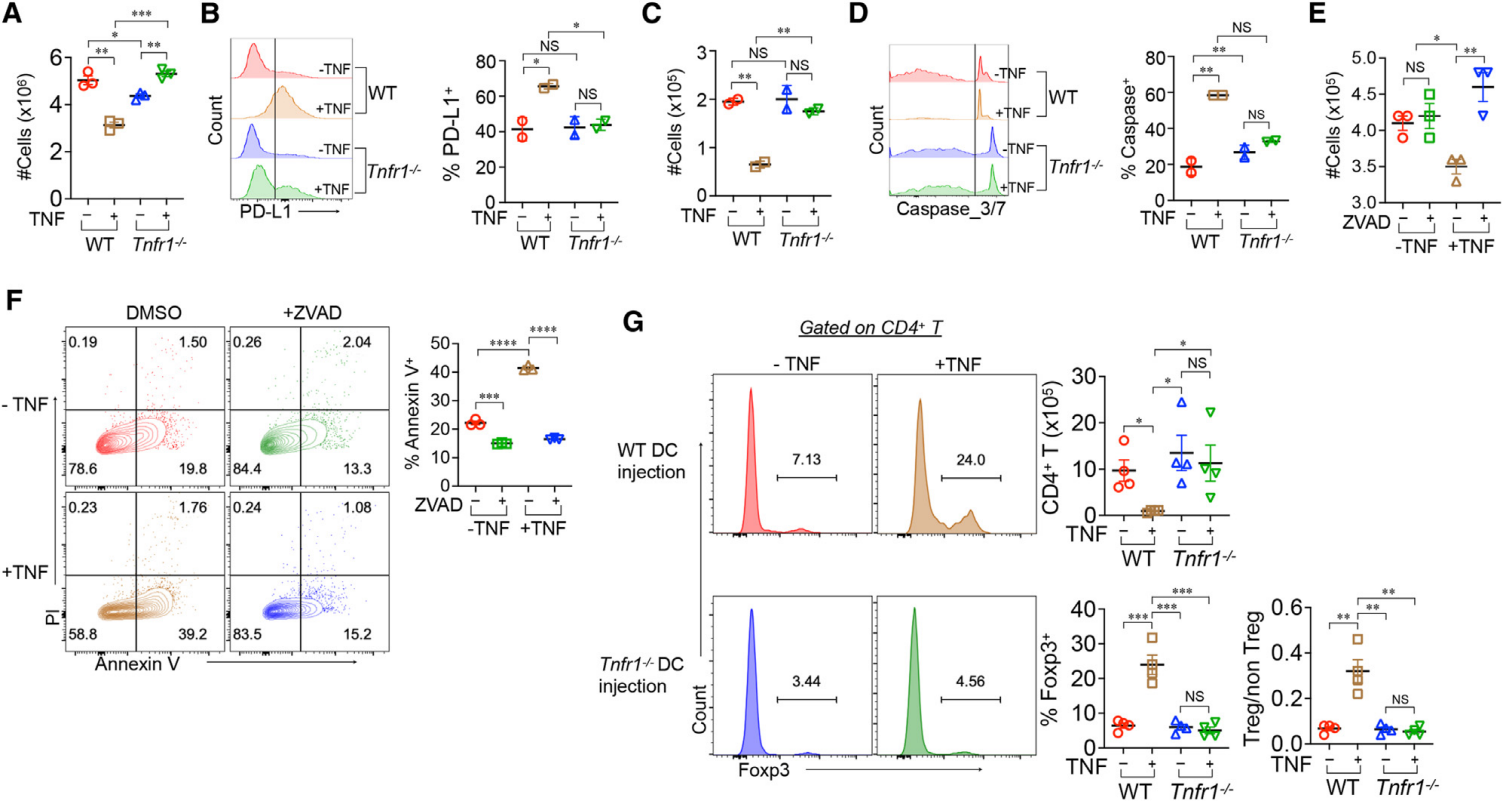
Effect of TNF on WT and TNFR1-deficient BMDCs (A and B) Bone marrow cells from WT or TNFR1-deficient mice were cultured with GM-CSF in the presence or absence of TNF for 5 days (BMDCs). Cells were counted by light microscopy (A) and PD-L1 expression on CD11c^+^ cells was determined by flow cytometry (B). (C) After 5 days, BMDCs were cultured for another 48 h in the presence of TNF or medium alone and live cells counted (C) and active caspase-3/7 measured by flow cytometry (D). (E and F) After day 5, BMDCs were cultured for an additional 48 h under the indicated conditions live cells (E) and apoptosis measured by staining with annexin V and propidium iodide (PI) (F). (G) DCs were generated from either OT-II or OT-IIx*Tnfr1*^*−/−*^ bone marrow as in A, pulsed with OVA whole protein overnight, washed, and 2 × 10^6^ cells intratumorally injected into OT-II KPC tumor-bearing mice. Three days after injection, tumor-infiltrating cells were isolated and analyzed. ∗*p* < 0.05, ∗∗*p* < 0.01, ∗∗∗*p* < 0.001, ∗∗∗∗*p* < 0.0001. NS, not significant.

To determine whether the consequences of anti-TNFR1 treatment *in vivo* were due to a direct effect on DCs themselves, we asked whether TNF affects the differentiation of BM precursors to DCs, and if so via which receptor. BM cells from WT or TNFR1-deficient mice were cultured with granulocyte-macrophage colony stimulating factor (GM-CSF) or Flt3L for 5 days in the absence or presence of TNF. The addition of TNF to WT BM inhibited DC generation (Figure 7A; Figure S7A) and, as previously shown,^33^ induced much higher levels of PD-L1 compared to GM-CSF or Flt3L alone (Figures 7B and S7B). In contrast, culture of TNFR1-deficient BM with TNF resulted in more DCs than GM-CSF or Flt3L alone, which presumably was due to signaling via TNFR2 (Figure 7A). Unlike WT BM, TNF had no effect on PD-L1 in the absence of TNFR1 (Figures 7B and S7B). PDAC tumors have been reported to have higher bacterial abundance, and antibiotic treatment has improved clinical outcome in patients receiving gemcitabine.^34^ Therefore, we tested whether TNF can induce PD-L1 on DCs in the presence of LPS and found results similar to those using non-activated DCs (Figure S7C). The effect of TNF on DC number could be due to the inhibition of differentiation and growth or an increase in cell death. To test the latter, BM-derived DCs (BMDCs) were generated from WT or TNFR1-deficient BM and then cultured with or without TNF in the presence of lipopolysaccharide (LPS), CpG, or medium alone. TNF caused the apoptotic death of WT but not TNFR1-deficient BMDCs, as determined by a large reduction in their number (Figure 7C, left panel) accompanied by activation of caspase-3 (Figure 7D, middle and right panel). Similar results were obtained when the BMDCs were activated with CpG or LPS (Figures S7D and S7E). TNF-induced apoptosis was prevented by the pan-caspase inhibitor ZVAD, as determined by cell recovery (Figure 7E) and annexin V staining (Figure 7F).

### TNF impairs DC ability to activate antigen-specific T cells in the tumor microenvironment

The finding that TNF elevated PD-L1 and decreased IL-12 production by DCs suggests that TNF-conditioned DCs would be poor antigen-presenting cells. To evaluate antigen-specific T cell responses in the TNF-rich PDAC microenvironment, we injected KPC cells subcutaneously into αβTCR transgenic OT-II mice, whose T cells are overwhelmingly CD4^+^ and recognize ovalbumin (OVA) peptide 323–339 presented by I-A^b^.^35^ Once tumors were established, they were injected on two consecutive days with 2 × 10^6^ OVA-pulsed WT or TNFR1-deficient BMDCs that had been generated in the presence or absence of TNF. Tumors were harvested 2 days after the second BMDC injection and infiltrating cells were analyzed. Transfer of TNF-conditioned WT but not TNFR1-deficient BMDCs resulted in half as many CD45^+^ tumor-infiltrating cells as medium-conditioned BMDCs (Figure S7F). This was primarily due to a marked reduction in the number of αβ T cells (Figure 7G). Moreover, as observed with irradiated KPC mice reconstituted with *Tnfr1*^*−/−*^ BM (Figure 3G), among the small number of T cells in the tumors receiving TNF-conditioned BMDCs, the fraction of Tregs was much higher than in controls. Therefore, BMDCs that survive in the presence of TNF acquire immunoregulatory characteristics that limit antigen-specific T cell accumulation and function in PDAC.

### TNFR1 expression inversely correlates with DC numbers in human PDAC

The mouse models of PDAC revealed that TNFR1 signaling decreased DC number and inhibited their function in the tumor. To ask if the relationship between TNFR1 signaling and PDAC DC infiltration observed in mice correlates with the human disease, we first analyzed publicly available data from The Cancer Genome Atlas Program (database: https://www.cancer.gov/ccg/research/genome-sequencing/tcga). Higher expression of TNFR1 in PDAC tumor tissue, as determined by bulk RNA sequencing, correlated with poorer survival (Figure S8A). Furthermore, analysis of scRNA-seq datasets of PDAC-infiltrating cells^36^ found that higher TNFR1 expression in myeloid cells inversely correlated with the number of DC1s and DC2s (Figure S8B). We used quantitative real-time PCR to quantify the expression of *TNFRSF1A* (TNFR1) and *TNFRSF1B* (TNFR2) in neoplastic tissues collected from 35 patients with PDAC. There were no differences in PDAC patient clinical parameters between the groups with lower (<1, median age 64) and higher (>1, median age 72) TNFR1 expression (Figure S8C). The absolute levels of *TNFRSF1A* and *TNFRSF1B* varied considerably and had no relationship to the levels of *XCR1* or *SIRPA*. However, when the data were segregated by the ratio of *TNFRSF1A* to *TNFRSF1B*, fewer DC1s (less *XCR1*) were found when the ratio favored *TNFRSF1A* (Figure S8D). A ratio of >1 also correlated with higher expression of PD-L1 (*CD274*). The correlation of this inverse relationship and fewer intratumor DC1s was confirmed by histopathological staining (Figure S8E). These data suggest that as in mice, TNF contributes to human PDAC dysfunctional anti-tumor immunity by suppressing DCs.

## Discussion

PDACs are complex solid tumors with a low mutational burden and an inflammatory and immunosuppressive microenvironment.^37^ The desmoplastic stroma, with a collagen-rich extracellular matrix, abundance of CAFs, and hypovascularity, may limit access by immune cells and impair delivery of therapeutic drugs, resulting in aggressive tumor growth and drug resistance.^38^ Inflammation has been cited as one of the hallmarks of cancer due to its tumor-promoting effects.^39^^,^^40^ Cytokines and chemokines produced by inflammatory cells can induce tumor growth, stimulate angiogenesis, induce fibroblast maturation and migration, and promote metastatic spread via lymphatic networks.^40^ Additionally, intratumor immunosuppressive mediators produced by myeloid-derived suppressor cells (MDSCs), DCs, M2-macrophages, Tregs, or Bregs, glucocorticoids regenerated from inactive metabolites by the tumors themselves, and cell surface checkpoint molecules and their ligands such as PD-1, PD-L1, TIM3, and CTLA4 can all contribute to an ineffective anti-tumor immune response.^41^

TNF, a hallmark of inflammation and abundant in the PDAC microenvironment, has been shown to promote PDAC growth in mice^23^^,^^42^ and humans.^43^ Several mechanisms for this activity have been suggested. TNF induces NF-κB-dependent epithelial-mesenchymal transition (EMT) in PDAC,^44^ as well as endothelial-mesenchymal transition, an important source of CAFs, at least in part by downregulating the endothelial receptor tyrosine kinase TIE1.^23^ EMT is implicated in resistance of tumor cells to apoptosis and chemotherapy, acquisition of stem-like attributes, and increased invasiveness and metastasis.^45^ TNF also reprograms more differentiated “classical” pancreatic neoplastic cells to assume a “basal-like” aggressive state^25^ and promotes an immunosuppressive microenvironment by upregulating the expression of tumor PD-L1.^46^ A major pro-inflammatory signaling pathway downstream of TNFR1 is initiated by its activation of p38,^47^ and specific inhibition of T cell p38, which is activated via an MAPK-independent alternative pathway,^48^ inhibited PDAC growth and improved the survival of KPC mice.^24^ In line with this, blocking of IL-17A, a cytokine downstream of TNF,^29^ improved tumor burden in KPC mice.^12^

Despite such evidence, attempts to treat PDAC with TNF blockers have had mixed results. For example, blocking TNF with antibodies in mice inhibited pancreatic tumor growth and metastasis^25^^,^^42^ and showed promise in treating human PDAC xenografts.^43^ Despite this, TNF blockade with etanercept, a soluble fusion protein containing the TNF-binding domain of TNFR2, did not significantly enhance the efficacy of gemcitabine as a single agent in patients with advanced pancreatic cancer.^49^ Among the possible reasons for this is that TNF also signals via TNFR2, which has been shown to enhance anti-tumor immunity by inducing CD8^+^ T cell proliferation and function.^50^ In contrast, TNFR2 signaling in KPC cells was found to have a tumor-intrinsic growth-promoting role due to upregulation of NF-κB-dependent growth and survival pathways and increased tumor PD-L1 expression.^51^ Understanding the cell-specific biological consequences downstream of each TNF receptor is therefore highly relevant to the design of therapies to thwart TNF’s tumorigenic effects.

DCs are potent antigen-presenting cells, and their scarcity in the PDAC tumor microenvironment is a key reason for immune escape.^52^ Upon activation, DCs upregulate a large number of molecules that are essential for immune cell migration and activation, including pattern recognition receptors, chemokine receptors (e.g., CCR7), costimulatory molecules (e.g., CD80, CD86, and CD40), MHC class I and II, and cytokines (e.g., IL-12, IL-1β, and TNF).^53^^,^^54^ Low numbers of DCs have been observed in PDAC tumors in patients^52^ and KPC mice,^12^ and among a cohort of patients with PDAC, the level of circulating DCs positively correlated with better survival.^13^^,^^14^ Several factors in the PDAC microenvironment contribute to impaired DC function: tumor-derived TGF-β, IL-10, and IL-6 suppress DC survival and proliferation,^55^ MDSCs inhibit DC maturation,^55^ and a subset of immunosuppressive CD11b^+^ DCs can induce Treg generation and cytotoxic T cell (CTL) suppression.^56^ In addition, in PDAC tumors, DCs undergo loss or downregulation of antigen-processing and presenting molecules, such as the transporter for antigen presentation and human leukocyte antigen class I.^57^ To circumvent impaired DC function, attempts have been made to create DC vaccines by activating DCs bearing tumor-associated antigens with synthetic peptides or purified proteins, DNA, RNA or viruses, tumor lysates, or with tumor cells themselves.^58^ Such DC vaccines have shown some promise in clinical trials, improving survival by 4–16.5 months,^58^ but difference in expression of tumor antigens among patients and complications such as delayed type of hypersensitivity responses have limited these strategies. It may be that the goal of enhancing anti-tumor immunity by improving antigen presentation could be achieved by TNFR1 blockade without the problems associated with manipulating DCs *ex vivo*. Selective TNFR1 might be useful in other settings as well, as TNFR1 deficiency has been shown to enhance the maturation of DC and CD8^+^ T cell function in lymphocytic choriomeningitis virus and hepatitis virus infections.^59^^,^^60^

We and others found that DC2s are the predominant DC subset in mouse and human PDAC.^12^^,^^36^^,^^61^^,^^62^ GSEA found that lack of TNFR1 signaling resulted in their increased expression of cellular activation of antigen-processing pathways that secondarily lead to enhanced CD8^+^ T cell-mediated cytotoxicity. Although DCs are particularly effective at antigen cross-presentation to CD8^+^ T cells,^63^^,^^64^ moDCs and DC2s can also perform this function.^65^^,^^66^^,^^67^ The upregulation of a host of genes involved in antigen processing and presentation, such as *β2m*, *Calr, Tapbp*, and others, in TNFR1-deficient mice suggests that TNF may suppress this ability in tumor-infiltrating DC2s. Furthermore, TNFR1 deficiency likely increases intratumor DC number by inhibiting apoptosis, irrespective of DC subset. Although TNFR1 blockade in the absence of T cells resulted in increased intratumor DC numbers, they did not acquire an activated phenotype. This is likely largely because signaling via cell surface CD40 is a major mechanism for DC activation, and activated CD4^+^ T cells are the predominant source of its ligand, CD40L.^31^ We did observe a higher number of infiltrating Th17 cells after TNFR1 KO BM transfer, a helper subset with a pro-tumorigenic role in PDAC.^12^ However, the number of Th17 cells in the tumor microenvironment was exceedingly small and unlikely to have an impact in this model. Together, the results are consistent with a model in which TNFR1 blockade leads to intratumor DC accumulation because of decreased apoptosis, improved local antigen presentation and increased antigen-specific T cell activation, CD40-mediated positive feedback, and licensing of CD8^+^ T cell anti-tumor activity.

Antigen-presenting cells, including DCs, express the TNF receptor superfamily member CD40 on the cell surface.^68^ Engagement with its ligand CD40L (CD154), which is primarily expressed by activated CD4^+^ T cells, results in DC upregulation of MHC molecules, CD80 and CD86, and IL-12, all of which enhance antigen presentation and T cell activation.^31^ Agonistic CD40 antibodies mimic the activity of CD40L and can substitute for it in murine models of T cell-mediated immunity.^68^^,^^69^ Furthermore, agonistic anti-CD40 antibodies increase the effectiveness of DC vaccines by reducing T cell tolerance and enhancing antigen-specific cytotoxic T cell responses in tumor-bearing mice.^70^ In an orthotopic model of PDAC, anti-CD40 agonistic antibody treatment inhibited intratumoral IL-27 production from myeloid cells, a cytokine that correlates with poor patient outcome.^71^ Several preclinical models, including spontaneous KPC mouse model, found that the anti-CD40 agonists enhanced the effectiveness of chemotherapy.^70^^,^^72^^,^^73^^,^^74^ In human trials, chemotherapy combined with agonistic anti-CD40 has shown promising anti-tumor immune response in solid tumors such as melanoma,^75^ mesothelioma,^76^ and pancreatic cancer.^75^^,^^77^ Although a previous study using a KPC mouse variant reported that anti-CD40 plus Flt3L modestly increased MHC-II levels on tumor-infiltrating DCs,^12^ we found that this combination resulted in a substantial reduction. The reason for the difference is unclear, but may be due to differences in the tumor models tested, the tumor genetics, or some other unknown variables. In any case, our finding that agonistic anti-CD40 in combination with Flt3L upregulated DC surface PD-L1 may limit the efficacy of such therapy. Although also resulting in activated DCs, anti-TNFR1 actually had the opposite effect on PD-L1, which may explain at least in part why it was more effective in inhibiting KPC cell growth.

Although antibodies targeting cell surface molecules in some cases mediate their biological effects *in vivo* by inducing antibody-dependent cellular cytotoxicity (ADCC) and/or antibody-dependent cellular phagocytosis (ADCP), we think they are an unlikely explanation for the efficacy of the anti-TNFR1 hamster IgG1 used in this study. First, TNFR1 is widely expressed, including on cells of the immune system, yet injection of anti-TNFR1 antibodies resulted in increased numbers of tumor-infiltrating hematopoietic and immune cells. Second, ADCC is mediated primarily by natural killer cells^78^ and ADCP by phagocytic cells,^79^ which are both present in *RAG2*^*−/−*^ mice. However, anti-TNFR1 treatment effect had no measurable effect on tumor growth compared to control. Finally, the results obtained with antibody blockade mirrored those obtained with genetic ablation of TNFR1. Together, these observations make it highly likely that it was inhibition of TNFR1 signaling and subsequent enhancement of adaptive immunity, not antibody-dependent innate responses, that accounted for inhibition of tumor growth.

As of this writing, there are five Food and Drug Administration-approved “TNF blockers” in clinical use. They include anti-TNF antibodies and antibody derivatives (infliximab [Remicade], adalimumab [Humira], certolizumab pegol [Cimzia], and golimumab [Simponi]) and a TNF decoy receptor (etanercept [Enbrel], a fusion protein containing the TNFR2 TNF-binding domain coupled with the Fc portion of human IgG1).^80^ A number of clinical trials have explored their efficacy in the treatment of cancer, and the results in PDAC trials have not been promising. When TNF blockers were used with the widely used gemcitabine, they were found to be safe but without therapeutic benefit compared to gemcitabine alone.^49^^,^^81^ As pointed out in the latter, this may have been due to the advanced state of the cancers and insufficient TNF blockade, because we found that when non-tumor cells in the tumor microenvironment cannot produce TNF, treatment with agonistic anti-CD40 and Flt3L was very effective and actually eliminated established KPC cell tumors. Therefore, a combination of “pro-DC” therapies, such as Flt3L plus anti-CD40 accompanied by TNF or TNFR1 blockade, might be a more effective strategy.

### Limitations of the study

The TNFR1 KO mice lack its expression in all tissues. We were able to determine that the effect of TNFR1 signaling on PDAC was mediated by hematopoietic cells, but we cannot formally determine that DCs are the primary target. Experiments with tissue-specific Cre and floxed *Tnfrsf1a* will be required for this. Furthermore, the *in vivo* experiments use a hamster anti-TNFR1 antibody, and an anti-hamster response may attenuate the response to prolonged treatment. Murinization of these antibodies might provide a better therapeutic reagent.

## STAR★Methods

### Key resources table

**Table:** 

REAGENT or RESOURCE	SOURCE	IDENTIFIER
**Antibodies**		

CD11c Monoclonal Antibody (N418), FITC	Invitrogen	Cat# 11-0114-82 RRID: AB_464940
CD45.2 Monoclonal Antibody (104), PerCP-Cyanine5.5	Invitrogen	Cat# 45-0454-82 RRID: AB_953590
CD40 Monoclonal Antibody (HM40-3), eFluor™ 450	Invitrogen	Cat# 48-0402-82 RRID: AB_2574017
MHC Class II (I-A/I-E) Monoclonal Antibody (M5/114.15.2), APC-eFluor™ 780	Invitrogen	Cat# 47-5321-82 RRID: AB_1548783
CD197 (CCR7) Monoclonal Antibody (4B12), Alexa Fluor™ 700	Invitrogen	Cat# 56-1971-82 RRID: AB_657687
CD274 (PD-L1, B7-H1) Monoclonal Antibody (MIH5), PE-Cyanine7	Invitrogen	Cat# 25-5982-82 RRID: AB_2573509
CD45R (B220) Monoclonal Antibody (RA3-6B2), PE-Cyanine7	Invitrogen	Cat# 25-0452-82 RRID: AB_469627
TCR beta Monoclonal Antibody (H57-597), Alexa Fluor™ 700	Invitrogen	Cat# 56-5961-82 RRID: AB_2802349
CD4 Monoclonal Antibody (GK1.5), APC-eFluor™ 780	Invitrogen	Cat# 47-0041-82 RRID: AB_11218896
CD8b Monoclonal Antibody (eBioH35–17.2 (H35–17.2)), eFluor™ 450	Invitrogen	Cat# 48-0083-82 RRID: AB_11218504
IFN gamma Monoclonal Antibody (XMG1.2), FITC	Invitrogen	Cat# 11-7311-82 RRID: AB_465412
TNF alpha Monoclonal Antibody (MP6-XT22), eFluor™ 450	Invitrogen	Cat# 48-7321-82 RRID: AB_1548825
CD103 (Integrin alpha E) Monoclonal Antibody (2E7), eFluor™ 450	Invitrogen	Cat# 48-1031-82 RRID: AB_2574033
CD24 Monoclonal Antibody (M1/69), PE-Cyanine7	Invitrogen	Cat# 25-0242-82 RRID: AB_10853806
F4/80 Monoclonal Antibody (BM8), Alexa Fluor™ 700	Invitrogen	Cat# 56-4801-82 RRID: AB_2574503
Ly-6G Monoclonal Antibody (1A8-Ly6g)	Invitrogen	Cat# 17-9668-82 RRID: AB_2573307
Brilliant Violet 650™ anti-mouse/rat XCR1 Antibody	BioLegend	Cat# 148220 RRID: AB_2566410
PE anti-mouse CD172a (SIRPα) Antibody	BioLegend	Cat# 144012 RRID: AB_2563550
Anti-Mouse CD120a (TNFR1) (Clone 55R-170) – Purified *in vivo* PLATINUM™ Functional Grade	Leinco Technologies	Cat#T950 RRID: AB_2832124
Armenian Hamster IgG Isotype Control – Purified *in vivo* PLATINUM™ Functional Grade	Leinco Technologies	Cat#T376 RRID:AB_2894150
InVivoPlus anti-mouse CD40	BioXCell	Cat#BP0016-2 RRID: AB_1107601
InVivoPlus rat IgG2a isotype control, anti-trinitrophenol	BioXCell	Cat#BP0089 RRID:AB_1107769
InVivoMAb recombinant Flt-3L-Ig (hum/hum)	BioXCell	Cat#BP0342 RRID:BE0342
InVivoPlus anti-mouse PD-1 (CD279)	BioXCell	Cat#BP0273 RRID:AB_2687796

**Biological samples**		

Human PDAC tissues	University Medical Center Mainz, JGU-Mainz, 55131 Mainz, Germany	N/A

**Chemicals, peptides, and recombinant proteins**		

LIVE/DEAD™ Fixable Blue Dead Cell Stain Kit, for UV excitation	Invitrogen	Cat#L34962
CellEvent™ Caspase-3/7 Green Flow Cytometry Assay Kit	Invitrogen	Cat#C10427
Recombinant Mouse TNF-α (carrier-free)	BioLegend	Cat#575204
Recombinant Mouse GM-CSF (carrier-free)	BioLegend	Cat#576304

**Deposited data**		

Single cell sequencing raw data	NCBI Gene Expression Omnibus (https://www.ncbi.nlm.nih.gov/geo/)	[Database]: [GSE260964]

**Experimental models: cell lines**		

KPC cell line	In house generated	#95775

**Experimental models: organisms/strains**		

C57BL/6	The Jackson Laboratory	Cat#000664 RRID: IMSR_JAX:000664
B6.129S7-*Rag1*^*tm1Mom*^/J	The Jackson Laboratory	Cat#002216 RRID: IMSR_JAX:002216
Rag2-Model RAGN12 (Constitutive knock out)	Taconic Biosciences	N/A
KPC (Kras^G12D/+^; p53^R172H/+^; PDX1-Cre) mice	Center for Advanced Preclinical Research, NCI	N/A
*Tnfr1*^*−/−*^	Gift from Zheng-Gang Liu, NCI	N/A
*Tnfr2*^*−/−*^	Gift from Joost Oppenheim, NCI	N/A

**Software and algorithms**		

BD FACSDiva software	BD Biosciences	RRID: SCR_001456
FlowJo	BD Biosciences	RRID: SCR_008520
GraphPad Prism 10	GraphPad Software	RRID: SCR_002798

### Resource availability

#### Lead contact

Further information and requests for resources and reagents should be directed to and will be fulfilled by the contacts, Jonathan D. Ashwell (jda@pop.nci.nih.gov), Muhammad S. Alam (alamms@mail.nih.gov).

#### Materials availability

All unique reagents generated in this study are available from the lead contact upon request.

#### Data and code availability

• Single-cell RNA-seq data have been deposited at GEO and are publicly available as of the date of publication. Accession numbers are listed in the key resources table.
• This paper does not report original code.
• Any additional information required to analyze the data reported in this paper is available from the lead contact upon request.

### Experimental model and study participant details

#### Mice

WT B6 (C57BL/6) (Stock#000664) mice were purchased from the Jackson Laboratory. *Rag2*^*−/−*^ (Rag2-RAGN12) mice were obtained from Taconic Biosciences. *Tnfr2*^*−/−*^ mice were obtained from Joost Oppenheim, Center for Cancer Research, National Cancer Institute, NIH, Frederick, MD,^82^ *Tnfr1*^−/−^ mice from Zheng-Gang Liu, Center for Cancer Research, National Cancer Institute, NIH, Bethesda, MD.^83^ KPC mice (Kras^G12D/+^; p53^R172H/+^; PDX1-Cre carrying oncogene Kras^G12D/+^; p53^R172H/+^; PDX1-Cre) were obtained from Center for Advanced Preclinical Research, Frederick National Laboratory for Cancer Research, NIH. All single constituent alleles of the KPC model have been crossed for at least 15 generations onto a standardized C57BL/6 background prior to intercrossing to assemble a tri-allelic model. All mice were maintained in a National Cancer Institute (NCI) specific pathogen-free animal facility, and all animal experiments were performed under an NCI Animal Care and Use Committee–approved animal study protocol.

#### Cell lines

The KPC cell line 95775 (mycoplasma-free) was generated from a B6-KPC tumor bearing mouse. The murine pancreatic cancer B6 cell line Panc02 was a gift from Jack Greiner, NCI; mycoplasma-free). Cells were grown in DMEM containing 10% fetal calf serum supplemented with penicillin/streptomycin and L-glutamine (complete medium) at 37°C in 5% CO_2_.

#### PDAC patients

Tissue samples were provided by the tissue bank of the University Medical Center Mainz in accordance with the regulations of the tissue biobank and the approval of the ethics committee of the University Medical Center Mainz. The study was approved by the ethics committee of the University of Mainz (statement code: 2019–14390; Landesärztekammer Rheinland-Pfalz), and written informed consent was obtained from all patients. The clinical parameters analyzed included local tumor extent/size (T stage), regionary lymph node metastasis (N stage), distant organ metastasis (M stage), tumor cell invasion in lymphatic vessels (L stage) or blood vessels (V stage), perineural tumor invasion (Pn stage), the presence of tumor cells in the surgical resection margin (R stage), and histopathological grading (G stage).

### Method details

#### Adoptive T cell transfer

For adoptive transfer experiments, T cells were isolated from spleens and lymph nodes of WT, *Tnfr1*^*−/−*^*,* and *Tnfr2*^*−/−*^ with a purity of ≥90%. 10^6^ cells/200 μL PBS were injected into the tail veins of recipient mice. After 2 weeks, 8 × 10^5^ KPC cells were inoculated in the right flank and tumor growth was monitored. Tumor size and burden did not exceed that allowed by the NCI animal study protocol LICB-054.

#### Isolation of tumor infiltrating T cells from subcutaneously inoculated tumors and intracellular staining

KPC tumors were explanted and minced into 1–2 mm pieces followed by incubation in RPMI (Gibco) containing DNase I (Roche) and Liberase (Roche) for 30 min. The digested tissue was pressed once through a 100 μm strainer and twice through a 70 μm strainer (BD Falcon) to create a single-cell suspension. After Percoll (Cytiva) density gradient separation, cells were stimulated with PMA/ionomycin in the presence of monensin at 37°C for 4 h. Cells were washed in FACS buffer (1% bovine serum albumin plus 0.1% sodium azide), stained for live/dead and cell surface markers for 30 min on ice, followed by washing with Perm/Wash solution for 30 min at 4°C. Cells were stained for 1 h at room temperature with antibodies and washed once with Perm/Wash solution, twice with FACS buffer, and analyzed by flow cytometry (FACS Fortessa). Data were analyzed by FlowJo 10.4 software.

#### Preparation of a single-cell suspension from pancreatic tumor tissues

Pancreatic tumors from treated and untreated KPC mice have been rapidly dissected upon euthanasia by CO_2_ asphyxiation. Single cell suspensions were prepared using Mouse Tumor Dissociation kit (Cat# 130-096-730, MACS Miltenyi Biotec) and a Gentle Macs Agitator (Miltenyi Biotec) following original manufacturer’s protocol. Briefly, dissected tumors have been cut into small fragments (1–2 mm^3^) and lysed in RPMI media supplemented with a proprietary cocktail of proteases for 1 h at 37^o^C with continuous agitation. Lysed tissues have been subsequently subjected to additional mechanic homogenization and final single cell suspensions prepared by filtering the homogenates through a 40 micron nylon cell strainer (Corning, Cat# CLS352340).

#### Survival study of KPC mice after bone marrow transfer

8-week-old KPC mice were treated with oral antibiotics for 1 week and then irradiated with 9 Gy. Mice were reconstituted with WT or TNFR1-deficient 10^7^ T cell–depleted (Dynabeads, Invitrogen) BM cells from WT or DKI mice. Mice were monitored for survival.

#### Single cell sequence data analysis

Barcodes filtration was done using miQC.^84^ Individual sample normalization was done using SCTransform through Seurat V3^85^ Doublets were detected using DoubletFinder V2 with a doublet estimate of 3% followed by data integration using the same package with default settings, clustering, dimension reduction and sample integration was performed using the Seurat V3 Package. Clustering was performed using the SLM algorithm and a resolution of 0.6 and projected using Uniform Manifold Approximation and Projection (UMAP)^86^ using the top 50 principal components (PCs). Average gene expression per cluster was calculated and clusters were annotated using SingleR^87^ with the https://www.immgen.org dataset in addition to manual inspection for CD4 and CD8α clusters. DC subsets were chosen based on SingleR annotation and manual inspection then using a resolution of 0.1 and top 30 PCs. Differential expression was performed using the FindMarkers function in Seurat with the options pseudocount.use = 0.5 and test.use = “MAST”,^88^ which uses a hurdle model for handling the zero inflated single cell data. GSEA analysis was performed by -log10 multiplied by *p*-value then multiplied by fold change sign from the differential expression analysis then fed into fGSEA.^89^

#### Real-time PCR

Dendritic cells from KPC tumor single cell suspensions were purified using CD11c^+^ cell isolation kit (Miltenyi Biotech Inc.). Total RNA was isolated using Qiagen RNeasy Plus mini Kit and was reverse-transcribed using Omniscript RT kit (Qiagen) according to the manufacturer’s instructions. Power SYBR Green premix (Applied Biosystems) was used for quantitative PCR. All data were normalized to GAPDH (glyceraldehyde-3-phosphate dehydrogenase) RNA and are presented as expression relative to this control. The primers were:

*IL-12B*-Fwd: 5′- GACATTCTGCGTTCAGGTCCAG -3′, *IL-12B*-Rev: 5′- CATTTTTGCGGCAGATGACCGTG-3′; *GAPDH*-Fwd: 5′- CATCACTGCCACCCAGAAGACTG-3′, *GAPDH*- Rev: 5′- ATGCCAGTGAGCTTCCCGTTCAG-3.

#### Treatment of subcutaneous and spontaneous KPC tumors

##### Subcutaneous

Mice (WT or *Tnfr1*^*−/−*^) were subcutaneously inoculated with 8 × 10^5^ cells in 50 μL PBS and allowed to grow for 2 weeks. Treatment with antibodies were performed by intratumor injection with different dose and frequencies: control Ab (2 times a wk, dose: 200 μg/injection), anti-TNFR1 (2 times a wk, dose: 200 μg/injection), anti-PD-1 (2 times a wk, dose: 100 μg/injection), anti-CD40 agonistic antibody (once in every 5 days, dose: 100 μg/injection), and/or Flt3L (every day for 5 consecutive days, dose: 30 μg/injection followed by 3 days interval before next round of 5 consecutive injection). Tumor growth was monitored over time. Except the survival study, tumor infiltrating cells were analyzed.

##### Spontaneous

KPC mice were screened by ultrasound and those with established tumors were divided in different groups depending on the experiments. When detected for tumor presence in the pancreas (day0/d0), mice were intraperitoneally treated with antibodies with similar frequencies and doses. Treatment continued until the mice get sick or reach human endpoint. At the end of the study, histopathology of the for H&E stain and DC staining.

#### Generation of BMDC and apoptosis assay

Bone marrow cells were flashed out with PBS, lysed with ACK lysing buffer (Cat#BP10-548E, Lonza) and cultured with 50 ng/mL GM-CSF for 5 days. Cells were further cultured for 48 h in the presence or absence of TNF in unstimulated, LPS (1 μg/mL), CpG (1 μg/mL) stimulated condition. Active caspase3_7 was measured by CellEvent Caspase-3/7 Green Flow Cytometry Assay Kit using the manufacturer instruction.

#### RNA isolation and quantitative real-time PCR

50-100 sections (5 μm) of cryopreserved human tissue were prepared using a microtome (cryostat), lysed with TRI Reagent Solution (Invitrogen), and RNA isolated according to the manufacturer’s instructions. cDNA was synthesized from 2 μg per sample with the High-Capacity cDNA Reverse Transcription Kit (Applied Biosystems) and then diluted with nuclease-free water. For quantitative real-time PCR, the SYBR Green PCR Master Mix (Applied Biosystems) was used. The reaction was performed using the QuantStudio 3 System (Applied Biosystems). Following primers were used: *RNA18S* forward 5′-CATGGCCGTTCTTAGTTGGT-3′, *RNA18S* reverse 5′-ATGCCAGAGTCTCGTTCGTT-3′, *XCR1* forward 5′-GATTCAGATGCTCTAAACGTC-3′, *XCR1* reverse 5′-AGAAACACCAGGCAGTATAG-3′, *CD1C* forward 5′-GATGTATGTACACAGGCAAG-3′, *CD1C* reverse 5′-CAGCATTAGGAAGAATATCACC-3′, *IL12B* forward 5′-AGAAAGATAGAGTCTTCACGG-3′, *IL12B* reverse 5′-AAGATGAGCTATAGTAGCGG-3′, *SIRPA* forward 5′-GAACGGAACATCTATATTGTGG-3′, *SIRPA* reverse 5′-CATGCAACCTTGTAGAAGAAG-3′, *TNF* forward 5′-CTCAGCCTCTTCTCCTTC-3′, *TNF* reverse 5′-AGAAGATGATCTGACTGCC-3′, *ITGAE* forward 5′-GGTGGGAGAAGAATTTAAGAG-3′, *ITGAE* reverse 5′-CATGCTGATGATGTTGTACC-3′, *CD274* forward 5′-CTCCAAATGAAAGGACTCAC-3′, *CD274* reverse 5′-TCCCTTTTCTTAAACGGAAG-3′, *WDFY4* forward 5′-AAGTCAGGAAACAAAGTGTC-3′, *WDFY4* reverse 5′-AGAAGTGTGACTACAATCCTC-3′, *TNFRSF1A* forward 5′-GCCTAGACACTGATGACC-3′, *TNFRSF1A* reverse 5′-TGCTGTATTGCGCCTC-3′, *TNFRSF1B* forward 5′-AGCACTGGCGACTTC-3′, *TNFRSF1B* reverse 5′-ACAAGGGCTTCTTTTTCAC-3′, *PDCD1LG2* forward 5′-TATCTGAACCTGTGGTCTTG-3′, *PDCD1LG2* 5′-GAATTCTTGTTCAGAGTCCAG-3′.

#### Immunohistochemistry

For visualization of infiltrated XCR1 positive cells, immunohistochemistry was performed. Formalin fixed and paraffin embedded tissue was cut into 3 μm thick slides (*n* = 35). After deparaffinization and re-hydration, a heat-induced antigen retrieval was conducted using an acidic buffer for FOXP3 (35 min; pH 6.1; Dako EnVision, Glostrup, Denmark) or a basic buffer for XCR1 (20 min; pH 9.0; Dako EnVision), followed by with the primary antibodies for 30 min at room temperature. The used antibodies were mouse anti-human FOXP3 (1:200; abcam) and rabbit anti-human XCR1 (1:1000; Invitrogen). After incubation with a secondary antibody (Dako), the binding was visualized with DAB+ chromogen (Dako). Slides were digitalized using an automated slide scanner (Aperio AT2, Leica Biosystems, Nussloch, Germany) at 400× magnification. Visualization and counting were performed in two representative slide areas of 1 mm^2^ using QuPAth 0.4.2 software (GitHub).

### Quantification and statistical analysis

All values are presented as mean ± SEM or SD, as indicated. Statistical analysis was performed with GraphPad Prism 7 software. Statistical significance was determined with the unpaired t-test, and *p* < 0.05 was considered to be statistically significant.

## Supplemental information

Document S1. Figures S1–S8

Document S2. Article plus supplemental information
