# Supplementary material for: TNFR1 signaling promotes pancreatic tumor growth by limiting dendritic cell number and function
Source: Cell Rep Med. 2024 Aug 22;5(9):101696. doi: 10.1016/j.xcrm.2024.101696 (PMC11528236; doi:10.1016/j.xcrm.2024.101696)
Supplement: Document S1. Figures S1–S8 [file mmc1.pdf]

Cell Reports Medicine, Volume *5*

Supplemental information

TNFR1 signaling promotes pancreatic tumor growth by limiting dendritic cell number and function

Muhammad S. Alam, Matthias M. Gaida, Hagen R. Witzel, Shizuka Otsuka, Aamna Abbasi, Theresa Guerin, Abdalla Abdelmaksoud, Nathan Wong, Margaret C. Cam, Serguei Kozlov, and Jonathan D. Ashwell

Supplemental information

TNFR1 signaling promotes pancreatic tumor growth by limiting dendritic cell number and function

Muhammad S. Alam, Matthias M. Gaida, Hagen R. Witzel, Shizuka Otsuka, Aamna Abbasi, Theresa Guerin, Abdalla Abdelmaksoud, Nathan Wong, Margaret C. Cam, Serguei Kozlov, and Jonathan D. Ashwell

Figure S1

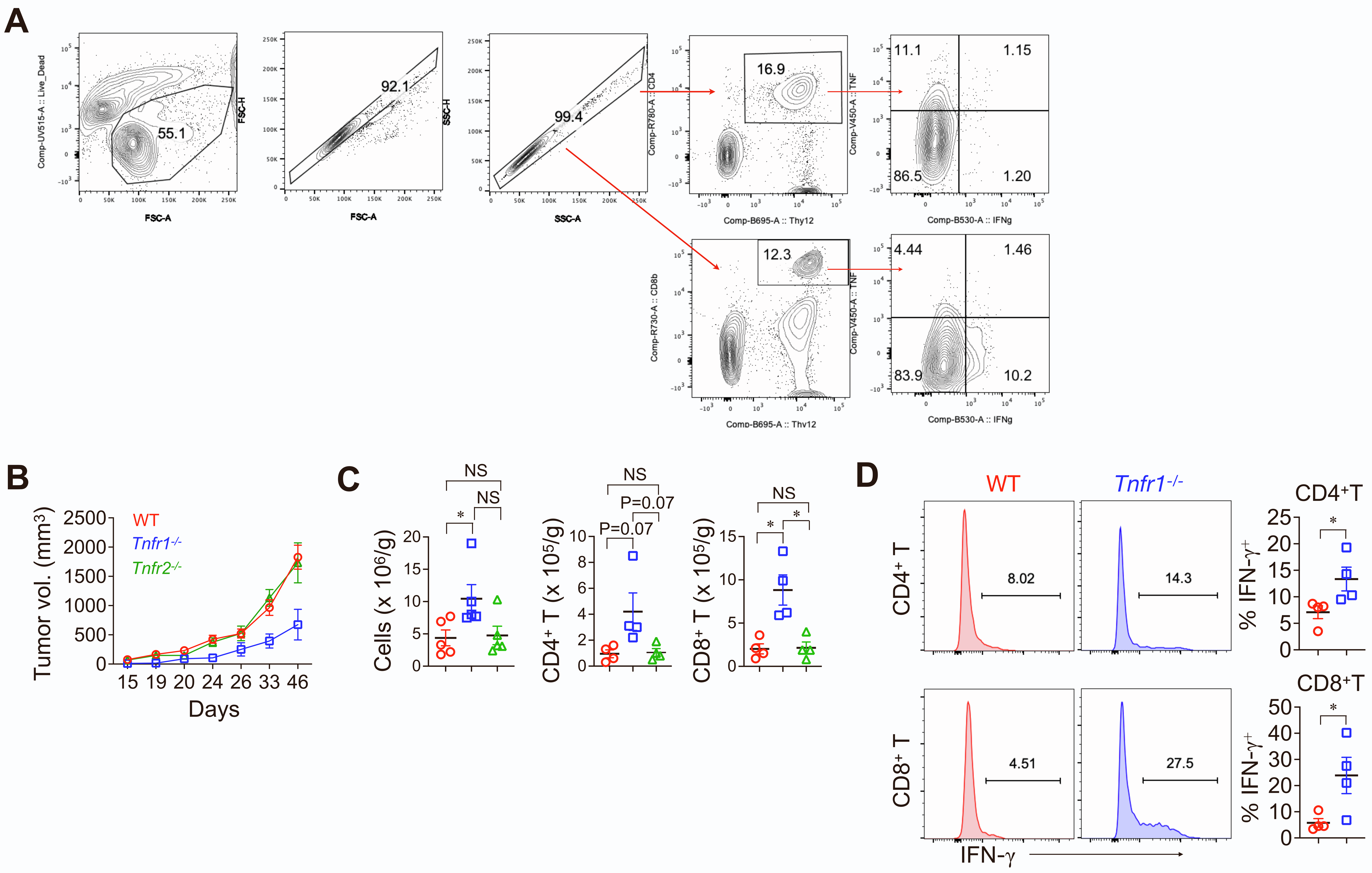

Figure S1. Analysis of Panc02 tumors. Related to Figure 1.

(A) Flow cytometry gating strategy. **(B-D)** Panc02 cells were implanted subcutaneously in WT (n=13), *Tnfr1-/-* (n=15), and *Tnfr2-/-* (n=9) mice, and tumor volumes measured over time **(B)**. At day 46, tumors were harvested, infiltrating cells were quantitated **(C)**, and infiltrating cells were stimulated with PMA/ionomycin before quantitation of T cell IFN-γ production **(D)**. *P<0.05, *P<0.01, ***P<0.001.

Figure S2

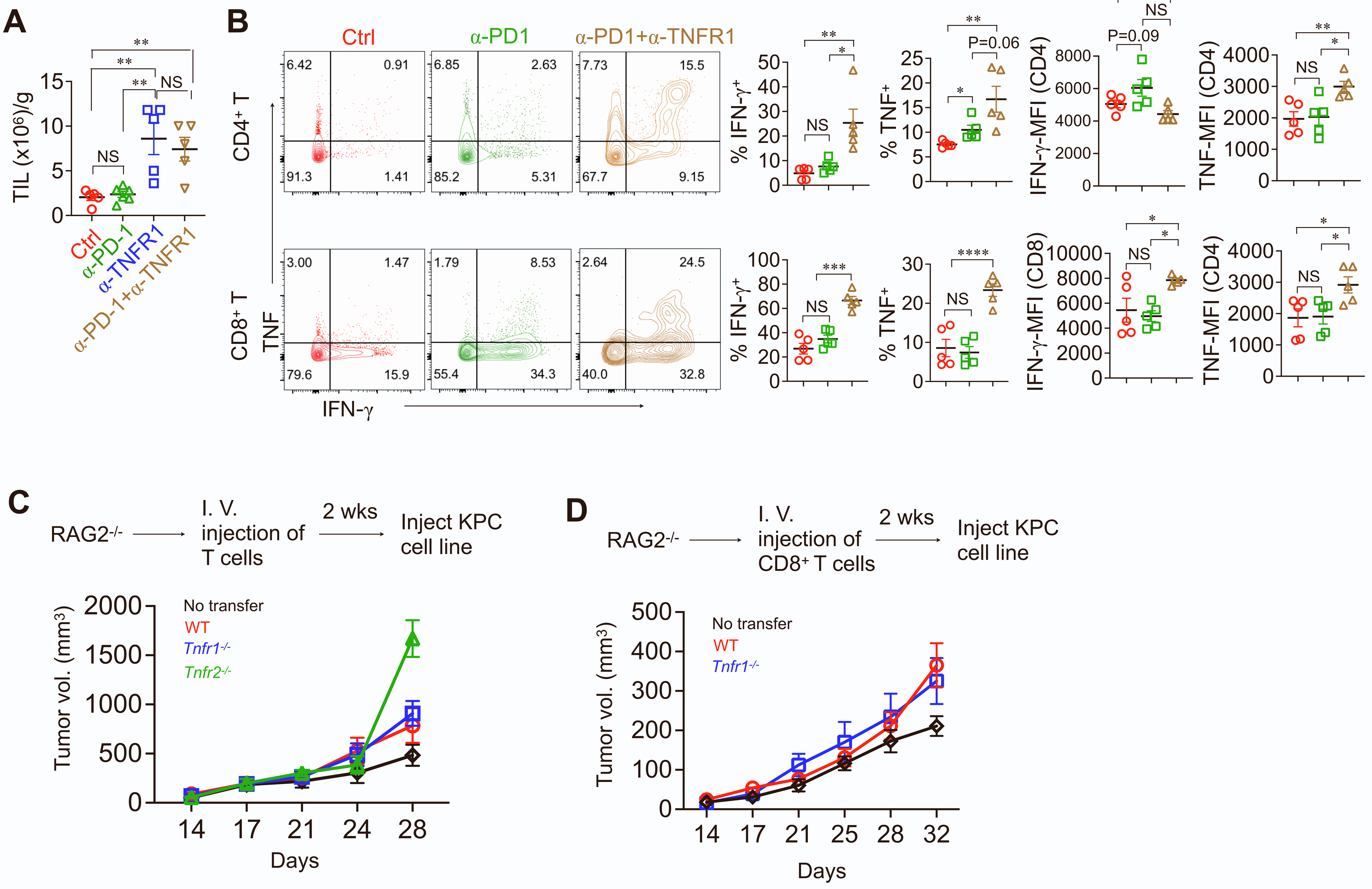

Figure S2. Analysis of tumor growth and infiltrating T cells after treatment of subcutaneous KPC tumor. Related to Figure 2.

(A-B) WT mice were subcutaneously injected with KPC cells that were allowed to grow for 2 wks. Mice were intratumorally injected with either control, anti-PD-1 (α-PD-1), anti-TNFR1 (α-TNFR1) or combination of both α-PD-1 and α-TNFR1 antibody (α-PD-1+α-TNFR1) and TIL was analyzed. **(A)** Total infiltrating cells as counted by hemocytometer. **(B)** WT mice were treated and TILs were stimulated with PMA/ionomycin and determined IFN-γ and TNF production in T cells by flow cytometry. **(C)** Total T cells from WT (n=7), TNFR1 knockout (n=8), or TNFR2 knockout mice (n=5) were injected into the tail vein of RAG2$^{-/-}$ mice. Another group was injected with PBS alone (n=4) (No transfer). Two wk later KPC cells were implanted subcutaneously. Tumor growth was monitored over time. **(D)** The same protocol as in panel C was followed except purified CD8^{+} T cells from WT (n=8) or TNFR1 knockout (n=7) mice were injected. Another group was injected with PBS alone (n=7) (No transfer). *P<0.05, *P<0.01, ***P<0.001, ****P<0.0001. NS=Not significant.

Figure S3

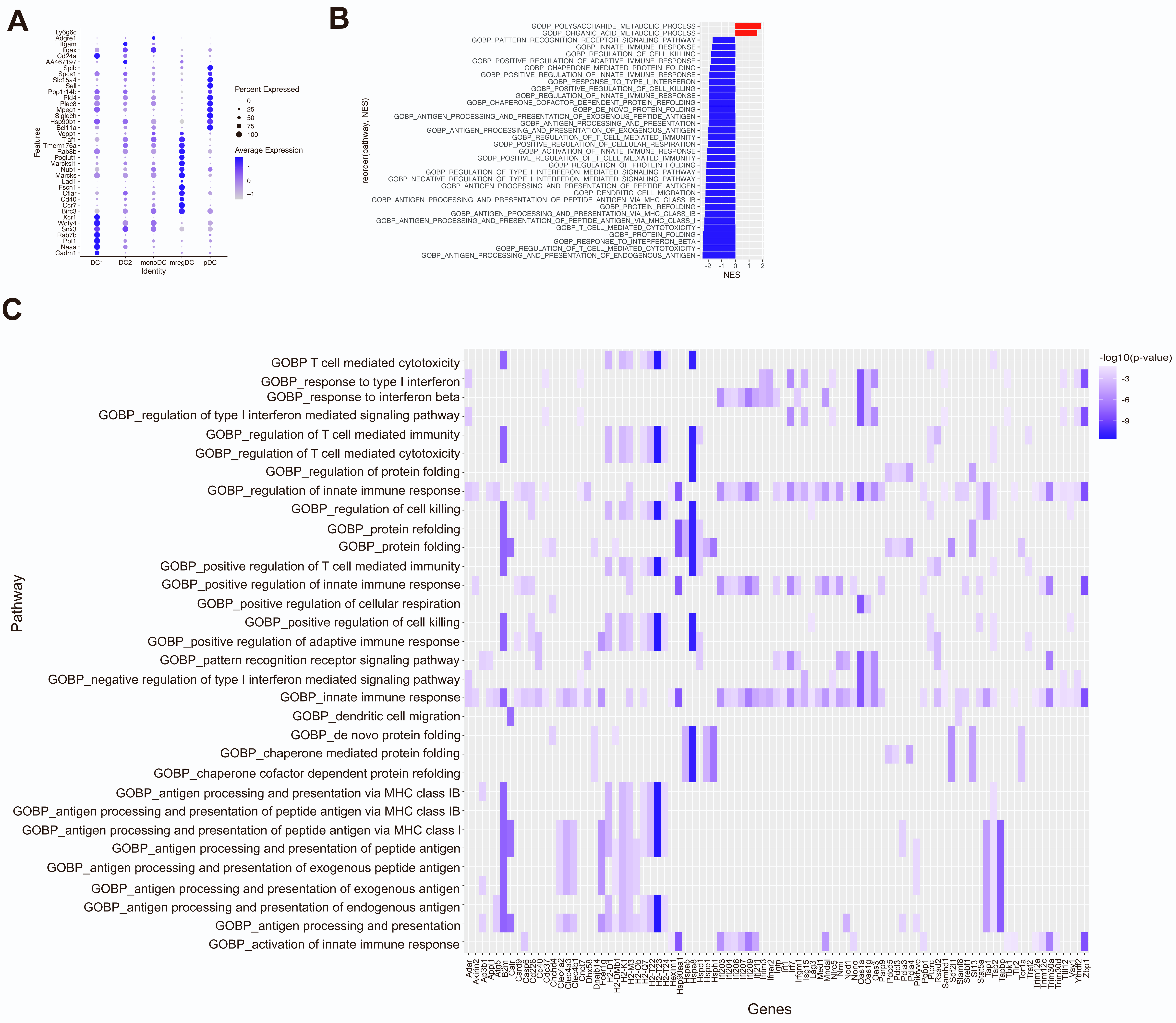

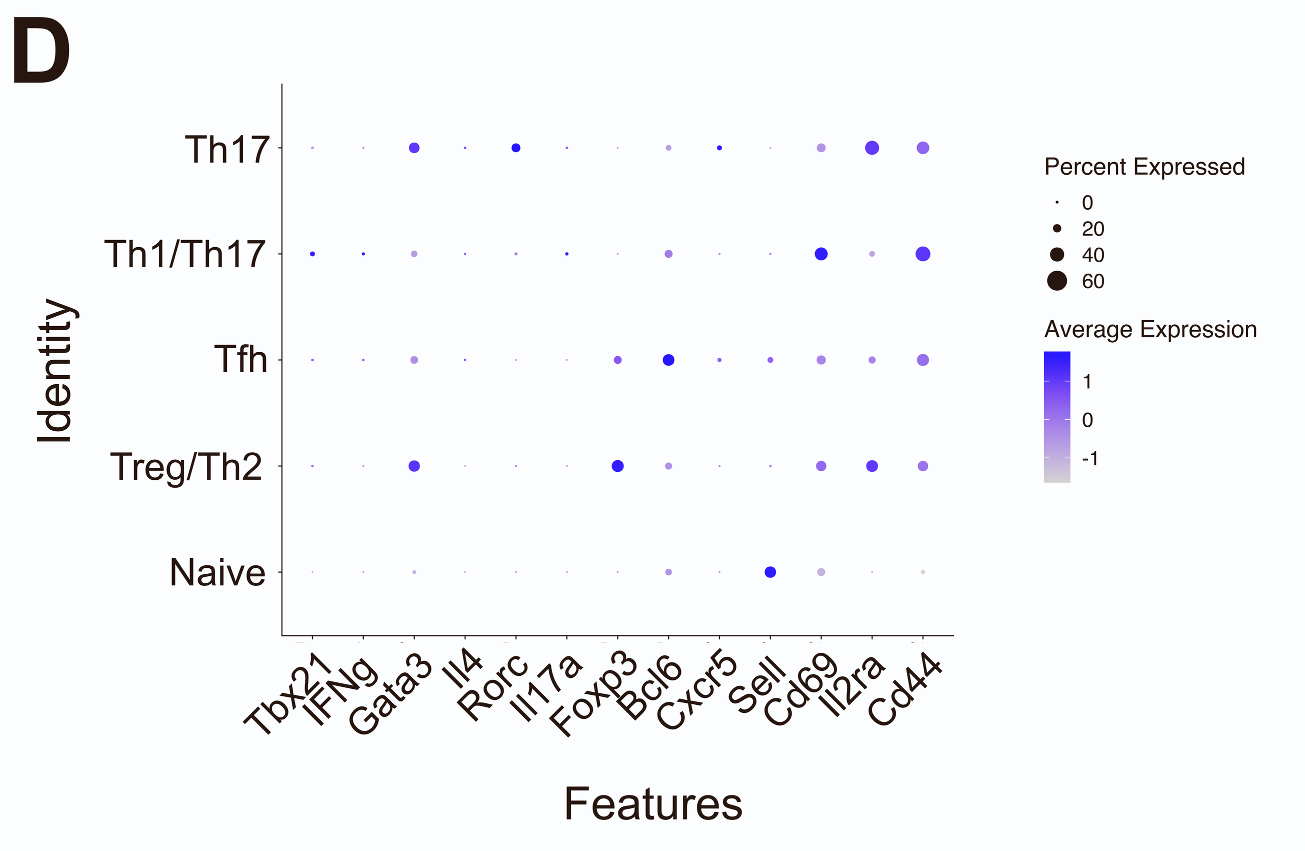

Figure S3. Characteristics of dendritic cells and CD4⁺ T cell subsets infiltrated in the pancreas of KPC mice after bone marrow transfer. Related to Figure 3.

KPC mice were lethally irradiated with 9 Gy at the age of 8 wk and transferred with either WT or TNFR1-deficient BM and analyzed tumor infiltrating dendritic cells by single cell RNA sequencing. (A) Gene signatures used to define different dendritic cell subsets. (B, C) GSEA of significantly differentiated pathways of dendritic cell activation/function (B) and their associated genes (C) are shown. D) Gene signatures used to define different CD4⁺ T cell subsets.

Figure S4

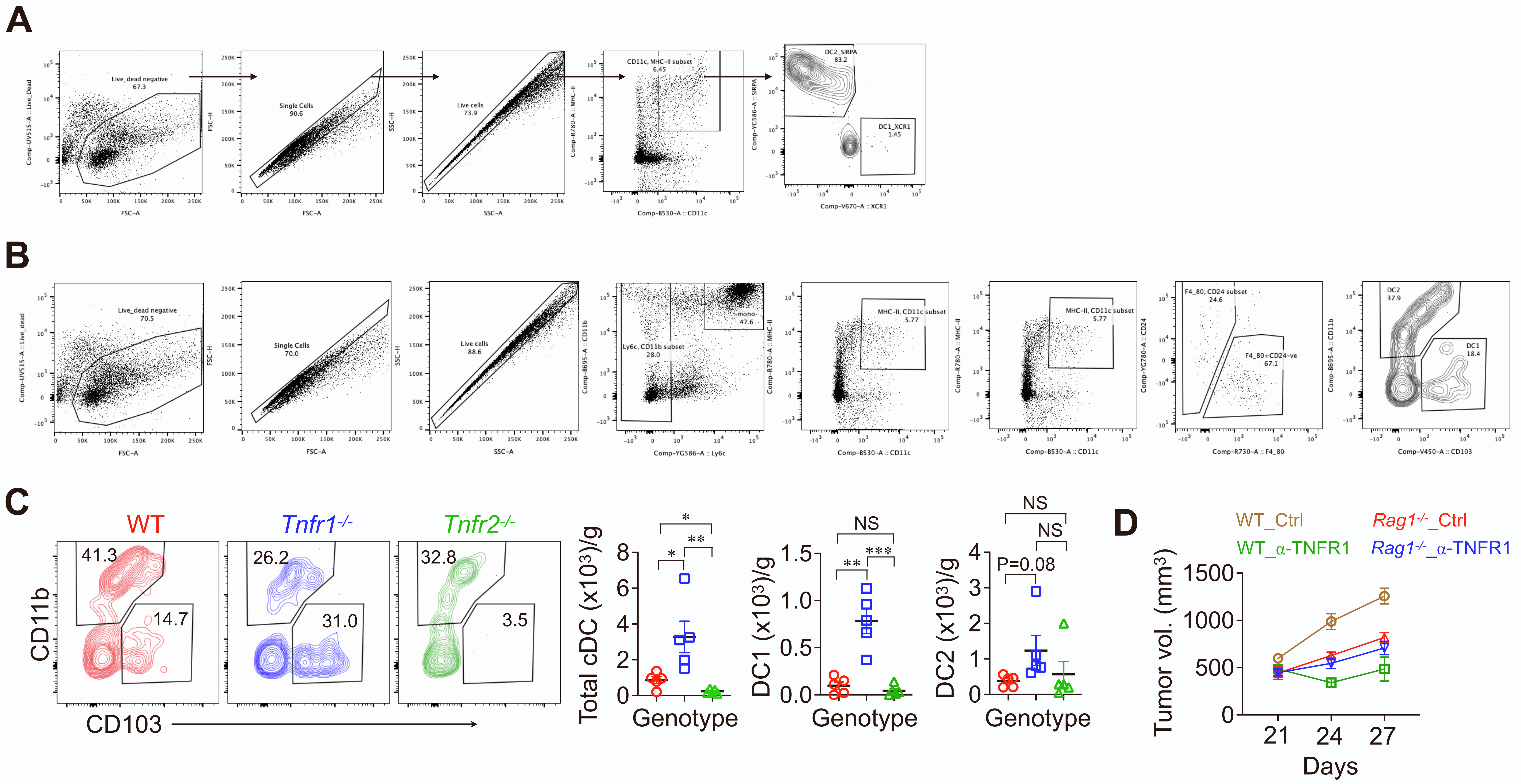

Figure S4. Dendritic cell infiltration in subcutaneous KPC tumors. Related to Figure 3.

(A) Gating strategy for Figure 4A. **(B, C)** KPC cells were subcutaneously implanted in the flank of WT (n=5), *Tnfr1*$^{-/-}$ (n=5), and *Tnfr2*$^{-/-}$ (n=5) mice. Tumor-infiltrating DC were analyzed. Gating strategies for DC **(B)** and number of tumor infiltrating DC subsets **(C)** are shown. **(D)** KPC cells were subcutaneously implanted in WT or *Rag-1*$^{-/-}$ mice, allowed to grow for 21 days, and then treated with control or anti-TNFR1 antibody every 3 days. Tumor volumes were followed over time. *P<0.05, **P<0.01, ***P<0.001. NS=Not significant.

Figure S5

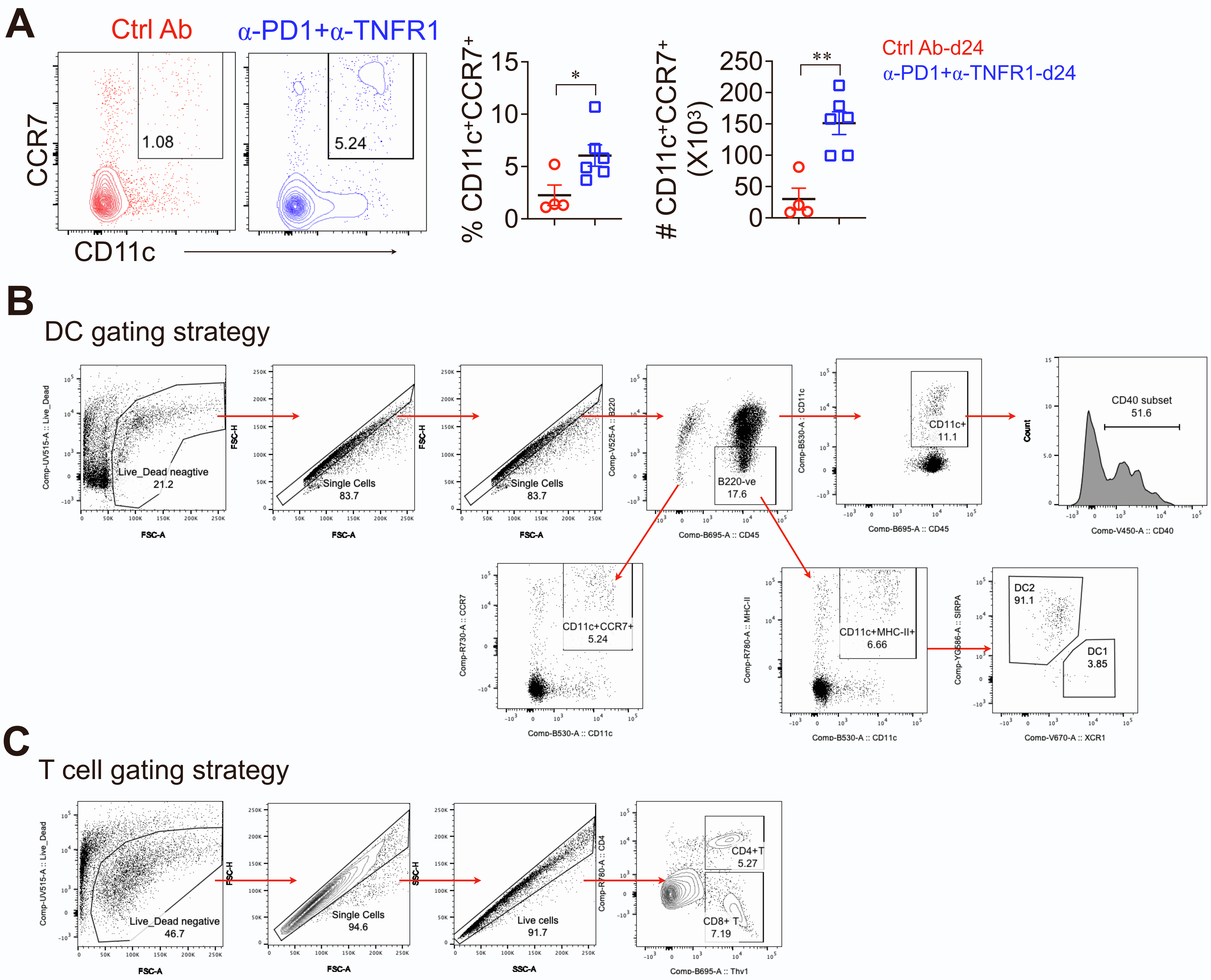

Figure S5. Blocking of both TNFR1 and PD-1 in KPC mice. Related to Figure 5.

(A) KPC mice were diagnosed for tumor in the pancreas by ultrasound and treated with either control or anti-TNFR1 plus anti-PD-1 antibody. Tumors were harvested at 24 and analyzed for DCs. **(B, C)** Gating strategy for DC **(B)** and T cells **(C)** are shown. **P*<0.05, ***P*<0.01. NS=Not significant.

Figure S6

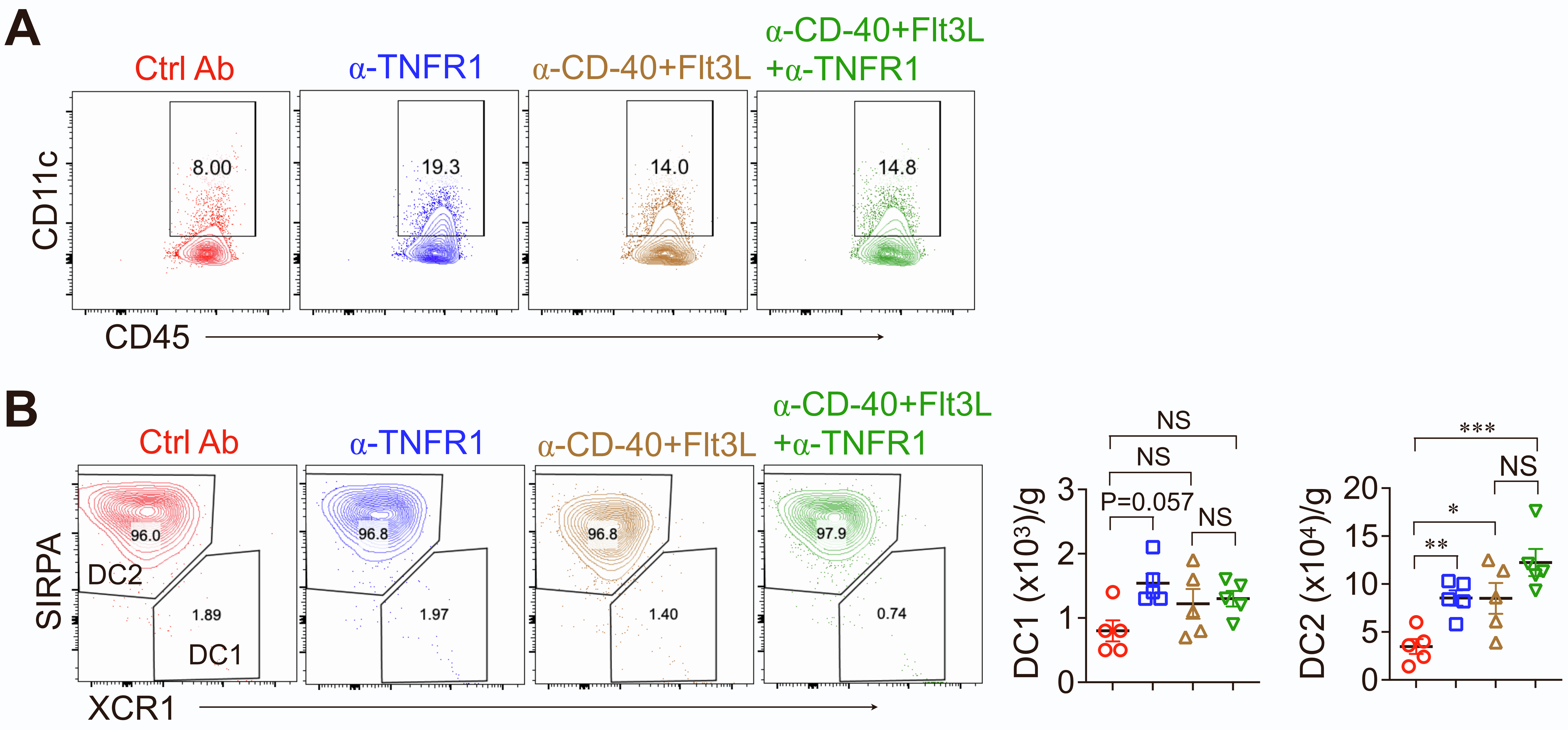

Figure S6. Dendritic cell subsets after treatment of subcutaneous KPC cell tumors with TNFR1 blockade with or without Flt3L and agonistic anti-CD40. Related to Figure 6.

Tumor-infiltrating cells from the tumor of the mice used in Figure 6 were stained for the indicated markers and analyzed by flow cytometry **(A)**. The DC subsets defined by these markers from multiple experiments are shown **(B)**. **P*<0.05, ***P*<0.01, ****P*<0.001, *****P*<0.0001. NS=Not significant.

Figure S7

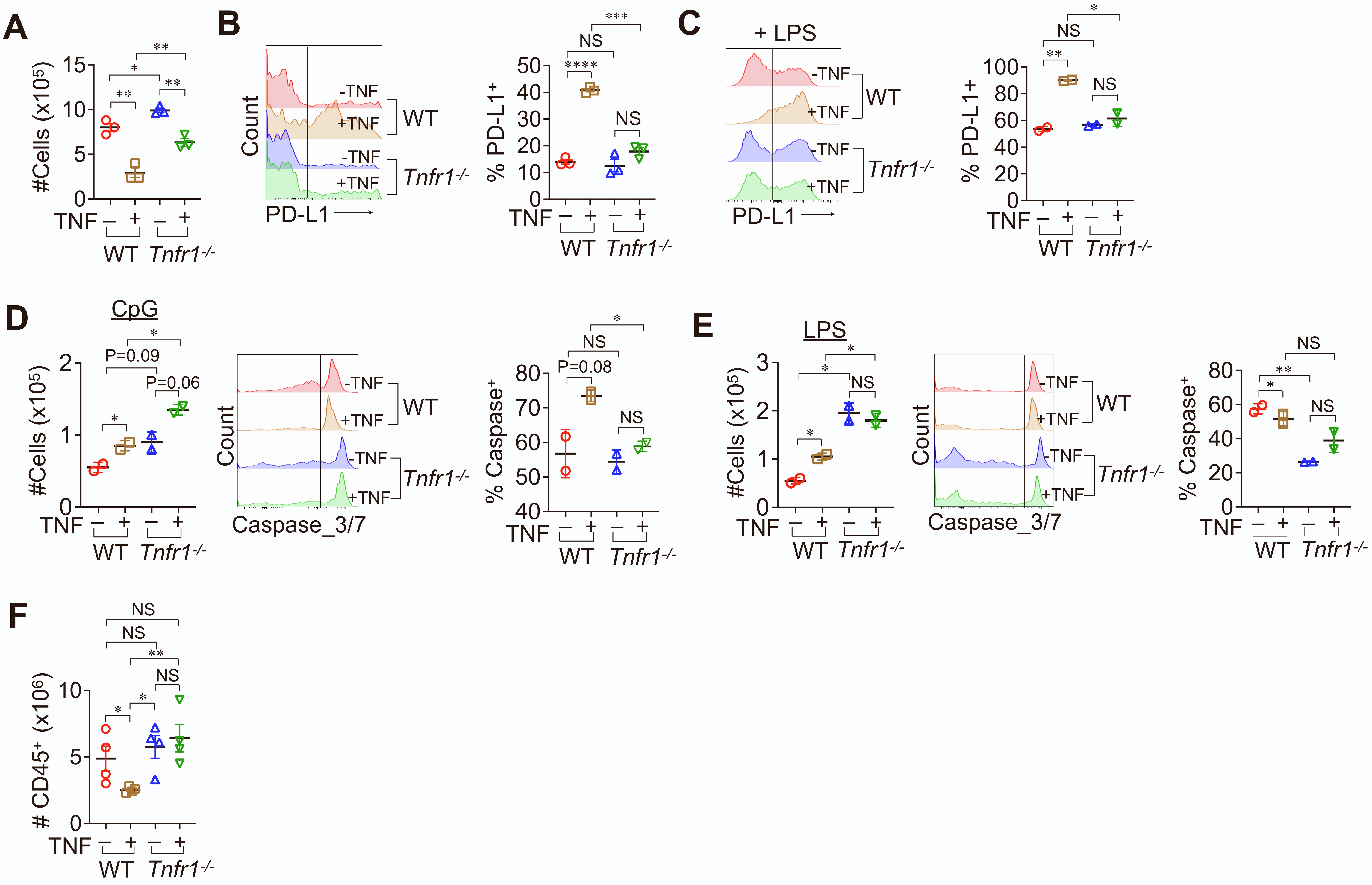

Figure S7. Effect of TNF on WT or TNFR1-deficient BMDC. Related to Figure 7.

(A, B) BMDC were generated from WT or TNFR1-deficient BM using Flt3L in the presence or absence of TNF for 5 days. Trypan-blue negative cells were counted by light microscopy **(A)**, and PD-L1 expression on $CD11c^+$ cells was determined by flow cytometry **(B)**. **(C)** BMDC were generated from WT or TNFR1-deficient BM using GMCSF in the presence or absence of TNF for 5 days. The cells were stimulated with LPS for 48 hr and PD-L1 expression on $CD11c^+$ cells is shown. **(D, E)** BMDC were generated from WT or TNFR1-deficient BM with GMCSF in the presence or absence of TNF for 5 days. DC were activated further with CpG **(D)** or LPS **(E)** for 48 h in the presence of TNF or medium alone, at which time the cells were counted and caspase-positive cells quantitated by flow cytometry. **(F)** DC were generated from either OT-II or OT-II x *Tnfr1-/-* BM with GMCSF in the presence or absence of TNF, pulsed overnight with OVA protein, washed with PBS and $2x10^6$ DC were intratumorally injected to OT-II tumor bearing mice. Three days after DC injection $CD45^+$ cells were quantitated. **P*<0.05, ***P*<0.01. NS=Not significant.

Figure S8

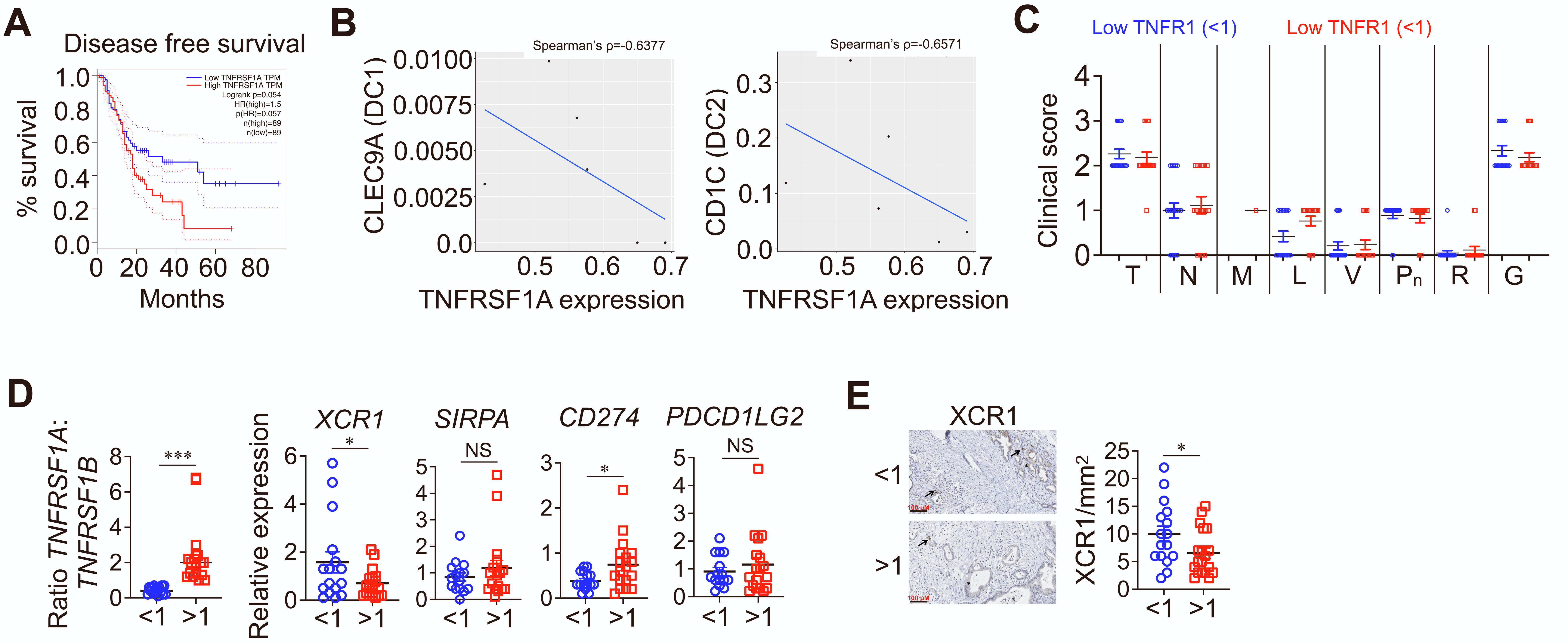

Figure S8. Correlations between human PDAC *TNFRSF1A* expression and *TNFRSF1A:TNFRSF1B* ratios with DC number and clinical prognosis.

Related to star methods (PDAC patient tissue and pathological classification).

(A) Disease-free survival of PDAC patients with high or low *TNFRSF1A* expression (TCGA data analyzed). **(B)** Correlation between *TNFRSF1A* expression in human PDAC-infiltrating myeloid cells and and expression of *CLEC9A* (DC1 marker) and *CD1C* (DC2 marker). Spearman's correlation (ρ) is shown in the top right corner. **(C)** Clinical parameters were evaluated between those with a ratio of <1 (n=19) and >1 (n=17). T=Local tumor extent/size, N=regionary lymph node metastasis, M=distant organ metastasis, L=tumor cell invasion in lymphatic vessels, V=tumor cell invasion in blood vessels, Pn=perineural tumor invasion, R=the presence of tumor cells in the surgical resection margin, and G=histopathological grading. **(D)** *TNFRSF1A* and *TNFRSF1B* were quantitated in human PDAC tumors (n=35) by RT-qPCR **(C)**. The samples were divided into two groups based on the ratio of *TNFRSF1A* to *TNFRSF1B*: < 1 (n=16) and > 1 (n=19). Expression of the indicated genes is shown for each group. **(E)** XCR1 protein-expressing cells (DC1 marker) were enumerated by immunohistochemistry and light microscopy, and the number of cells/ mm^2 as a function of the *TNFRSF1A:TNFRSF1B* ratio shown. *P<0.05, ***P<0.001. NS=Not significant.
